# Mechanisms of vagus nerve stimulation for the treatment of neurodevelopmental disorders: a focus on microglia and neuroinflammation

**DOI:** 10.3389/fnins.2024.1527842

**Published:** 2025-01-15

**Authors:** Makenna Gargus, Benneth Ben-Azu, Antonia Landwehr, Jaclyn Dunn, Joseph P. Errico, Marie-Ève Tremblay

**Affiliations:** ^1^Division of Medical Sciences, University of Victoria, Victoria, BC, Canada; ^2^Department of Pharmacology, Faculty of Basic Medical Sciences, Delta State University, Abraka, Nigeria; ^3^Vagus Nerve Society, Atlantic Beach, FL, United States; ^4^Department of Biochemistry and Molecular Biology, The University of British Columbia, Vancouver, BC, Canada

**Keywords:** vagus nerve, acetylcholine, microglia, neurodevelopmental disorders, vagus nerve stimulation

## Abstract

The vagus nerve (VN) is the primary parasympathetic nerve, providing two-way communication between the body and brain through a network of afferent and efferent fibers. Evidence suggests that altered VN signaling is linked to changes in the neuroimmune system, including microglia. Dysfunction of microglia, the resident innate immune cells of the brain, is associated with various neurodevelopmental disorders, including schizophrenia, attention deficit hyperactive disorder (ADHD), autism spectrum disorder (ASD), and epilepsy. While the mechanistic understanding linking the VN, microglia, and neurodevelopmental disorders remains incomplete, vagus nerve stimulation (VNS) may provide a better understanding of the VN’s mechanisms and act as a possible treatment modality. In this review we examine the VN’s important role in modulating the immune system through the inflammatory reflex, which involves the cholinergic anti-inflammatory pathway, which releases acetylcholine. Within the central nervous system (CNS), the direct release of acetylcholine can also be triggered by VNS. Homeostatic balance in the CNS is notably maintained by microglia. Microglia facilitate neurogenesis, oligodendrogenesis, and astrogenesis, and promote neuronal survival via trophic factor release. These cells also monitor the CNS microenvironment through a complex sensome, including groups of receptors and proteins enabling microglia to modify neuroimmune health and CNS neurochemistry. Given the limitations of pharmacological interventions for the treatment of neurodevelopmental disorders, this review seeks to explore the application of VNS as an intervention for neurodevelopmental conditions. Accordingly, we review the established mechanisms of VNS action, e.g., modulation of microglia and various neurotransmitter pathways, as well as emerging preclinical and clinical evidence supporting VNS’s impact on symptoms associated with neurodevelopmental disorders, such as those related to CNS inflammation induced by infections. We also discuss the potential of adapting non-invasive VNS for the prevention and treatment of these conditions. Overall, this review is intended to increase the understanding of VN’s potential for alleviating microglial dysfunction involved in schizophrenia, ADHD, ASD, and epilepsy. Additionally, we aim to reveal new concepts in the field of CNS inflammation and microglia, which could serve to understand the mechanisms of VNS in the development of new therapies for neurodevelopmental disorders.

## 1 Introduction

The vagus nerve (VN) is the primary component of the parasympathetic nervous system, regulating homeostatic functions throughout the body and brain (Agostoni et al., 1957). A key function of the VN is modulation of the inflammatory reflex, a systemic immune response that peripherally involves the spleen and the activation of choline acetyltransferase (ChAT)-positive (+) cells, i.e., the peripheral cholinergic anti-inflammatory pathway (Pavlov et al., 2003; Pavlov and Tracey, 2012). Additionally, VN activation influences the immune microenvironment of the central nervous system (CNS) through direct release of acetylcholine (ACh) from the nucleus basalis of Meynert (NB), which modulates microglia, the innate immune cells of the CNS (Hays et al., 2013; Nichols et al., 2011). The functional mechanisms of VN immunomodulation have been studied using vagus nerve stimulation (VNS), which uses electrical pulses to modulate VN activity. VNS is also an approved therapy for individuals with refractory epilepsy, treatment-resistant depression, and severe primary headaches (Dawson et al., 2021; Fisher et al., 2020; Silberstein et al., 2016). Given that CNS inflammation (or ‘neuroinflammation’) is associated with neurodevelopmental disorders, VNS may be effective as a therapeutic alternative. In this review we examine the effect of VNS on CNS inflammation, microglial states, and other changes in the microenvironment to explore possible mechanisms and applications of VNS for neurodevelopmental conditions, including schizophrenia, autism spectrum disorder (ASD), and attention-deficit hyperactivity disorder (ADHD).

## 2 Vagus nerve anatomy and physiology

The VN, also known as cranial nerve X, is the longest among the twelve paired cranial nerves (Agostoni et al., 1957). These emerge directly from the brain to innervate the head and neck as motor (efferent) nerves, sensory (afferent) nerves, or a combination of both (Agostoni et al., 1957; Goggins et al., 2022). The VN serves as a mixed sensory-motor nerve, comprising approximately 80% afferent and 20% efferent fibers (Foley and DuBois, 1937). Afferent fibers of the VN are primarily composed of small-diameter, unmyelinated C fibers, which conduct afferent visceral information slowly (Ruffoli et al., 2011). A smaller population of larger diameter A and B fibers conduct afferent visceral information, motor input, and parasympathetic input much faster (Ruffoli et al., 2011). These fibers are involved in involuntary reflexes, such as the cough and gag reflex, and the transmission of sensory information (Ruffoli et al., 2011). The efferent VN fibers originate from rootlets exiting from the dorsal motor nucleus of the vagus (DMV) and nucleus ambiguous (NA) in the ventral medulla oblongata (Ruffoli et al., 2011; Wiles et al., 2007). These are responsible for stimulation of branchial arch striated muscles and control of parasympathetic functions (Ruffoli et al., 2011).

Understanding the basic anatomy and physiology of the VN in both the brain and body is essential to grasping the full breadth of its many functions. The Latin word “vagus,” meaning “wandering,” is aptly applied to the VN due to its extensive and complex path of innervation throughout the body (Ruffoli et al., 2011). Upon exiting the base of the skull, the VN subsequently innervates structures of the head and neck such as the larynx and pharynx, and additionally sends fibers that make up the auricular branch of the VN (ABVN) to innervate the outer ear (Howland, 2014). The two sides of the VN asymmetrically innervate the heart, with the right VN specifically innervating the sinoatrial node, which is the heart’s pacemaker, while the left innervates the atrioventricular node (Ruffoli et al., 2011). In the thorax, the VN additionally innervates the lungs and esophagus that it follows down into the abdomen (Ruffoli et al., 2011). The VN extensively innervates the stomach, which is its largest source of sensory information, as well as numerous abdominal organs, with its furthest reaching fibers innervating the distal third of the transverse colon (Ruffoli et al., 2011).

**Figure 1:**
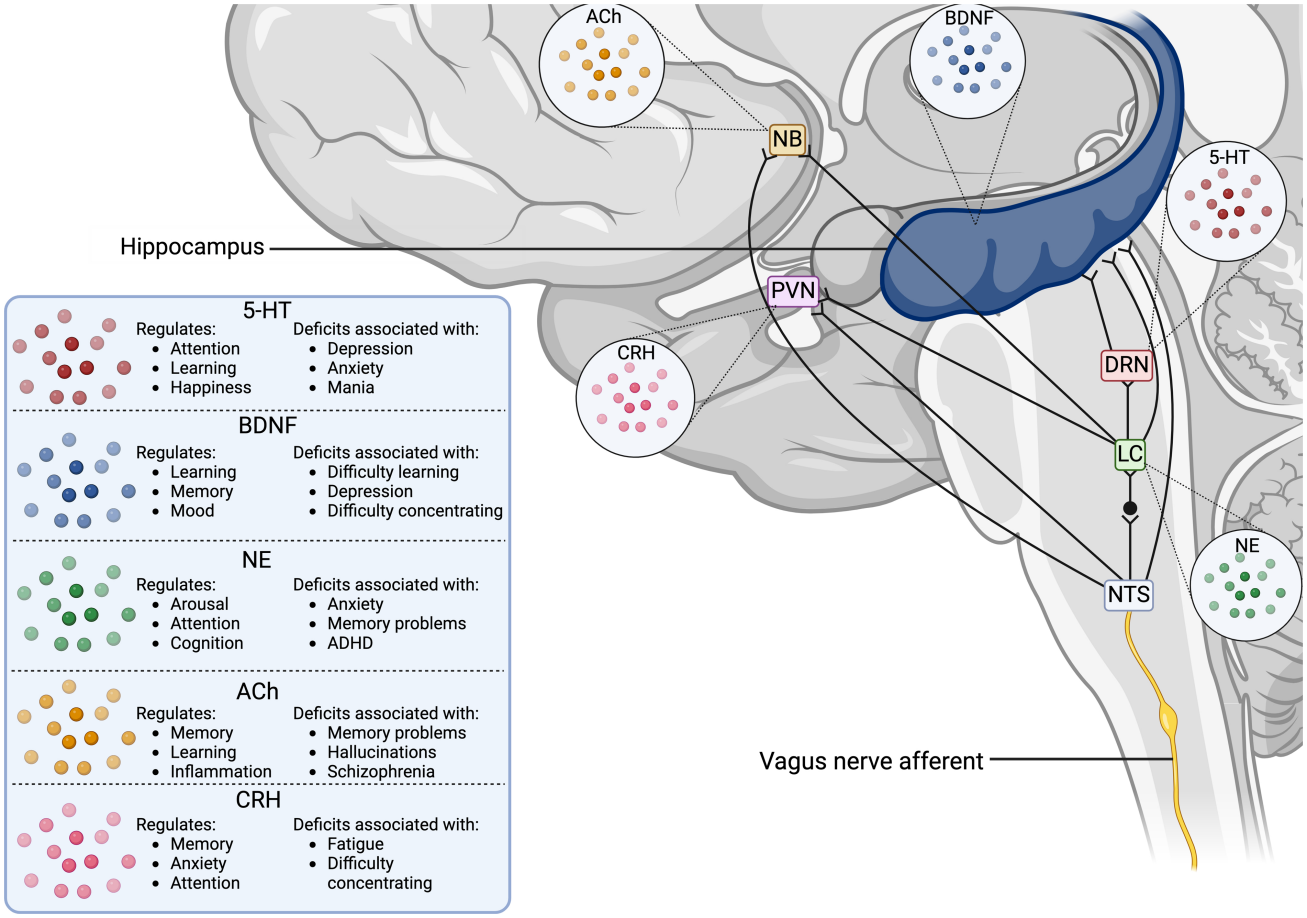
Vagus nerve neurotransmitter and synaptic pathways in the human brain. The illustration displays the direct synaptic connections from the incoming vagus nerve afferent signals to brain regions associated with neurotransmitter release. The table outlines the cognitive functions the stimulated neurotransmitters modulate, as well as the disorders associated with deficits in the neurotransmitter concentrations. 5-HT, serotonin; ACh, acetyl choline; BDNF, brain-derived neurotrophic factor; CRH, corticotropin releasing hormone; DRN, dorsal raphe nucleus; LC, locus coeruleus; NB, nucleus basalis of Meynert; NE, norepinephrine; NTS, nucleus tractus solitarius; PVN, paraventricular nucleus.

The VN collects peripheral information from its visceral branches and sends it through afferent projections to the tractus solitarius, which utilizes glutaminergic neurotransmission to synapse to the nucleus tractus solitarius (NTS) (Wang et al., 2021; Snell, 2010; Andresen and Yang, 1990). The NTS is a major sensory processing center in the brain that sends projections to numerous brain regions for further signaling (Figure 1), including major structures like the thalamus, hippocampus, rostral ventrolateral medulla, amygdala, and cerebral cortex (Breit et al., 2018). One important NTS projection connects to the NB, a region of the basal forebrain responsible for producing the neurotransmitter ACh for the prefrontal cortex, hippocampus, and amygdala (Hulsey et al., 2016). ACh is an important neurotransmitter that has been implicated in cognitive aspects like memory, attention, and motivation, as well as in regulating inflammation (Gombkoto et al., 2021). Stimulation of NB cells can modulate the release of ACh in its interconnected brain regions to enact a cholinergic response in the brain (Hays et al., 2013; Nichols et al., 2011; Ananth et al., 2023). The NTS can also modulate the NE concentrations through a di-synaptic pathway that connects the NTS to the locus coeruleus (LC), a region responsible for the production of norepinephrine (NE) (Manta et al., 2009a). NE activates α -adrenergic receptors ( α -ARs) and β -ARs in the brain, initiating increased arousal, attention, and the formation and retrieval of memories (Hays et al., 2013). The LC is part of the circuit that connects the VN to the serotonergic dorsal raphe nucleus (DRN), which enables the VN-mediated modulation of serotonin (5-HT) (Manta et al., 2009a). Chronic application of VNS in rats has demonstrated that prolonged production of NE can indirectly control the release of 5-HT in the DRN and ultimately increase 5-HT transmission in the hippocampus (Manta et al., 2009a). The LC is also known to send one-way connections to the NB that can excite cholinergic neurons to stimulate ACh release, establishing a direct link between the adrenergic and cholinergic systems in the CNS (Taylor et al., 2022). Projections from the NTS directly connect to the paraventricular nucleus (PVN), which is responsible for modulating corticotrophin-releasing hormone (CRH) as part of the hypothalamus-pituitary–adrenal (HPA) axis (Pavlov et al., 2003). The downstream effect of HPA axis stimulation is stress mediation through increased production of cortisol, a potent inflammatory inhibitor (Bonaz et al., 2017). This link between the NTS and PVN provides the VN a pathway to modulate the neuro-hormonal anti-inflammatory responses in the body (Pavlov et al., 2003). Additionally, the NTS harbors synaptic connections to the rostral ventrolateral medulla (RVM), which plays a role in cardiovascular homeostasis (Pavlov et al., 2003). Finally, the VN can mediate hippocampal functions and the production of brain-derived neurotrophic factor (BDNF) through connections arising from the NTS, LC, and DRN (Pavlov et al., 2003).

Vagal afferents also establish direct connections to the area postrema (AP) and DMV, which, along with the NTS, form the dorsal vagal complex (Pavlov et al., 2003). The DMV, serving as the motor component of the VN, receives processed NTS signals via gamma-aminobutyric acid (GABA) to regulate visceral functions through efferent signaling (Davis et al., 2004). Vagovagal reflexes, those mediating digestive functions, are controlled by inhibitory connections sent from the NTS to the DMV after afferent processing (Davis et al., 2004; Li and Owyang, 2003). In addition, the highly vascularized AP acts as a circumventricular organ, enabling the brain to have access to toxins, cytokines, and circulating hormones in the blood without crossing the blood–brain barrier (BBB), thus allowing for direct humoral immune-to-brain communication (Price et al., 2008). The AP possesses receptors for interleukin (IL)-1R1, which can induce c-Fos signals in the NTS and PVN, while IL-1 β -induced activation of the HPA axis also depends on the AP (Price et al., 2008).

## 3 The vagus nerve and the systemic immune system

The body’s innate immune system is a major component of immune responses, responsible for the defense against infection and injury (Pavlov and Tracey, 2004). Innate immune cells, such as granulocytes, macrophages, and dendritic cells, are activated by pathogen-associated and danger-associated molecular patterns received by pattern recognition receptors on the cell surface, including Toll-like receptors (TLRs) and nucleotide-binding oligomerization domain-like receptors (NLRs) (Pavlov et al., 2003; Pavlov and Tracey, 2012). As a result of downstream signaling cascades, there is an increased production and release of pro-inflammatory mediators like tumor necrosis factor (TNF), IL-6, and IL-1 β , which play critical roles in homeostatic immune response, such as extracellular pathogen clearance, neutrophil recruitment, and vasodilation (Pavlov and Tracey, 2004). Localized increases in TNF lead to common signs of inflammation, such as heat, swelling, pain, and redness in the skin (Pavlov and Tracey, 2004). The immune response is normally localized to the site of injury or infection, and is regulated by the release of anti-inflammatory mediators such as TGF β , IL-4 and IL-10 and through ACh signaling (Pavlov and Tracey, 2012). However, disturbances in the regulation and activity of the innate immune system can lead to chronic inflammation, caused by the continuous release of pro-inflammatory cytokines and decreased VN activity (Pavlov and Tracey, 2012). Chronic inflammation can then lead to the development of several diseases, including cardiovascular disease, diabetes mellitus, and neurodegenerative disorders (Furman et al., 2019). Additionally, inflammation during critical developmental periods, such as during pregnancy, infancy, and early childhood, can result in increased risk for neurodevelopmental disorders such as ASD, schizophrenia, and epilepsy (Jiang et al., 2018).

Immunomodulation via the VN is a crucial homeostatic function that relies on bidirectional communication between the brain and body. Afferent VN fibers detect immune imbalances and synapse the information to the NTS and its connected brain regions for processing, while efferent signals are generated within the CNS and sent to the DMV to conduct an immune response (Davis et al., 2004). Vagal motor neurons and efferent fibers originating from the DMV and NA then provide parasympathetic regulation to the body through the principle neurotransmitter ACh (Pavlov et al., 2003). Synthesis of ACh begins with a reaction between choline and acetyl coenzyme A, catalyzed by ChAT (Grando et al., 2003). The action of ACh is terminated by its hydrolysis by acetylcholinesterase or butyrylcholinesterase (Leuzinger et al., 1968). ACh acts on ionotropic nicotinic (nAChRs) and metabotropic muscarinic receptors (Gombkoto et al., 2021). Nicotinic receptors are ligand-gated ion channels with α - and β -subunits that form 12 subtypes ( α 2–10 and β 2–4), while muscarinic ACh receptors are G-protein-coupled receptors with five subtypes, divided into excitatory (M1, M3, and M5) and inhibitory (M2 and M4) receptors (Eickhoff et al., 2022). ACh plays an important role in immunomodulation, being released by immune cells, such as T cells, natural killer cells, and lymphocytes, as a response to infection (Suarez et al., 2018). ACh mediates concentration-dependent decreases in the pro-inflammatory mediator TNF and other pro-inflammatory mediators, such as IL-1 β , IL-6, and IL-18, through post-transcriptional mechanisms (Borovikova et al., 2000; Cox et al., 2020).

### 3.1 The humoral immune pathway

The sympathetic nervous system is complexly connected to the VN, and its parasympathetic functions and regulation of the autonomic nervous system can lead to functional and structural changes in the CNS. Environmental factors, such as psychological stress, diet, infection, and pollution, can influence the function of the VN in neurodevelopment and cognitive processes. Stress, for example, is a major modulator of VN function and excessive stress can lead to epigenetic alterations in synaptic structure and function (Murphy and Heller, 2022). In response to stress, the body activates the autonomic nervous system – comprised of the sympathetic and parasympathetic branches (Murphy and Heller, 2022). The HPA axis is a primary component of the sympathetic nervous system – the “fight or flight” response – and stimulates the secretion of glucocorticoids and catecholamines (Murphy and Heller, 2022). These hormones influence changes in emotional and arousal states, increase heart rate and blood pressure, decrease gut motility and secretion, and decrease bronchi diameter (Murphy and Heller, 2022). Once stress has been mitigated, the body returns to its homeostatic functions, as mediated by the parasympathetic nervous system – the “rest and digest” condition – which is primarily controlled by the VN (Murphy and Heller, 2022). However, in rodent models exposed to chronic stress, the autonomic nervous system can become dysregulated, leading to elevated glutamate levels and dendritic shrinkage in the CA1, CA3 and dentate gyrus regions of the hippocampus, as well as in the medial amygdala and prefrontal cortex (McEwen, 2017; Chaouloff et al., 2007). To prevent excessive activation and maintain homeostasis, the autonomic nervous system meets at nerve junctions (plexuses) to bridge communication between the sympathetic and parasympathetic systems (Howland, 2014). The VN can also directly communicate with components of the sympathetic nervous system in the CNS, such as the HPA axis, and indirectly connect to the sympathetic preganglionic neurons in the upper spinal cord (Pavlov et al., 2003). These CNS communication lines are crucial for the sympathetic and parasympathetic systems to work synergistically in humoral and VN-mediated parasympathetic immune responses throughout the body (Pavlov et al., 2003).

The humoral immune pathway for immune-to-brain communication relies on the HPA axis as the major component to initiate immune response. This pathway involves circulating cytokines, including TNF and IL-1 β , that cross the BBB and act on surface receptors on the brain capillary endothelium to enhance the release of prostaglandins, whose diffusion into the parenchyma mediates fever response and triggers HPA axis activation (Pavlov et al., 2003). Furthermore, the AP is a circumventricular organ that acts as a transduction site, allowing direct systemic signaling to the NTS and RVM, which further synapse to the HPA axis and sympathetic nervous system (Pavlov et al., 2003). The VN-mediated activation of PVN cells causes CRH to be synthesized and enter the pituitary portal system, where it stimulates adrenocorticotrophin hormone synthesis in the anterior pituitary, which in turn stimulates the release of cortisol from the adrenal cortex (Pavlov et al., 2003). The HPA axis is regulated through multiple negative feedback loops, such as the inhibition of CRH by adrenocorticotrophin hormone, and modulation by neural ACh, GABA, and 5-HT (Pavlov et al., 2003). Cortisol influences the inflammatory response by binding intracellular receptors and suppressing nuclear factor κ B activity (NF- κ B) expression, which is linked to pro-inflammatory cytokine synthesis (Pavlov et al., 2003). The sympathetic nervous system plays a role in both pro-inflammatory and anti-inflammatory processes through the LC and RVM, which connect to the sympathetic preganglionic cholinergic neurons in the spinal cord (Pavlov et al., 2003; Elenkov et al., 2000). These neurons then synapse with the postganglionic neurons, using NE as their primary neurotransmitter (Pavlov et al., 2003; Elenkov et al., 2000). During early inflammatory stages, the sympathetic nervous system can activate the inflammatory response at a local level through stimulation of α _2_-ARs (Pavlov et al., 2003; Elenkov et al., 2000). Activation of β -ARs on lymphocytes and macrophages by NE inhibits pro-inflammatory cytokine production via the β _2_-AR-cAMP-protein kinase A pathway and elevate anti-inflammatory cytokine levels (Pavlov et al., 2003).

### 3.2 The inflammatory reflex

The inflammatory reflex is a VN-mediated response to immune challenge comprised of two arms: afferent and efferent (Pavlov et al., 2003; Pavlov and Tracey, 2012). Peripheral pro-inflammatory molecules are received by afferent VN fibers, and signals are sent to the NTS, where they synapse to interconnected brain regions, such as the hypothalamic nuclei, amygdala, and insular cortex, to coordinate autonomic and endocrine responses (Pavlov et al., 2003; Pavlov and Tracey, 2012). One study demonstrated that intraportal administration of IL-1 β results in a dose-dependent increase in afferent activity in the hepatic branch of the VN in rats, which was not observed following hepatic vagotomy, therefore suggesting the presence of IL-1 β receptors on VN afferents (Niijima, 1996). Furthermore, the administration of IL-1 β resulted in a reflex activation of the sympathetic splenic nerve, which was similarly lacking in vagotomised rats (Niijima, 1996). Further studies implicated the participation of IL-1 β receptors in VN afferents and chemosensory cells in the paraganglia surrounding the afferent endings of the VN (Goehler et al., 1999; Ek et al., 1998). However, studies using the inflammogen lipopolysaccharides (LPS) via intraperitoneal injection or using intraperitoneal injection of IL-1 β in vagotomised rodents found that high levels of circulating IL-1 β could produce fever and sickness behaviors by bypassing neuronal circuits and acting directly on the brain through circumventricular organs like the AP, or other humoral mechanisms. Therefore, VN-mediated responses work in a dose-dependent fashion and appear especially important at early stages of infection, when circulating IL-1 β levels are low (Goehler et al., 2000; Hansen et al., 2001). Additionally, endocrine processes can be slower in comparison to neural regulation, emphasizing the crucial role of the VN and sympathetic nervous system in eliciting a rapid initial immunoregulatory response (Pavlov and Tracey, 2004). Signal integration in the NTS and associated brain regions, such as the PVN, RVM, and LC, creates the substrate for the HPA axis and sympathetic nervous systems to interact with the VN playing a central immunomodulatory role (Pavlov et al., 2003).

The efferent arm of the inflammatory reflex is constituted by the cholinergic anti-inflammatory pathway. When activated through the NTS-DMV synapse, the efferent signals travel to the celiac superior mesenteric ganglion complex, where they connect to the splenic nerve (Pavlov and Tracey, 2012). Stimulation of the splenic nerve by the efferent VN fibers leads to the release of NE in the spleen (Pavlov and Tracey, 2012). Splenic NE binds to β _2_-ARs found on the surface of memory CD4+ T cells expressing ChAT (Pavlov and Tracey, 2012). This subsequently triggers the synthesis and release of ACh from the T cells (Pavlov and Tracey, 2012). It was originally thought that ACh is directly released from the nerves, however studies found that the inflammatory reflex failed to inhibit TNF release in T cell-deficient nude mice, indicating that T cells play a role in the process (Rosas-Ballina et al., 2011). When T cells were repopulated in the T-cell deficient nude mice, the response was restored, suggesting direct signaling via NE due to the proximity of splenic lymphocytes to the adrenergic nerve endings (Rosas-Ballina et al., 2011). Together, these findings supported the role of ChAT T-cells in the ACh release required for the inflammatory reflex. Peripheral immune cells, such as macrophages, dendritic cells, and monocytes, are a major source of TNF, and the expression of α 7AChR in bone marrow-derived cells is essential for ACh regulation of TNF release (Pavlov and Tracey, 2012). The ChAT T-cell derived ACh binds α 7AChR to affect downstream signaling pathways, inhibiting NF- κ B nuclear translocation and activating the Janus kinase 2-signal transducer and activator of transcription 3 mediated signaling cascade (Pavlov and Tracey, 2012; de Jonge et al., 2005). This results in the inhibition of TNF transcription along with other pro-inflammatory mediators. A study using human macrophage cultures exposed to LPS demonstrated that ACh can inhibit pro-inflammatory cytokines without affecting the release of anti-inflammatory cytokines (Pavlov and Tracey, 2012; Borovikova et al., 2000). Electrical stimulation of the VN also effectively decreases serum TNF levels in wild-type mice, but is ineffective in mice lacking nicotinic receptors, further validating this important pathway (Pavlov et al., 2003).

## 4 Microglia, central nervous system inflammation, and the vagus nerve

The VN plays a complex role in influencing the systemic immune response and has an impact on the immune microenvironment within the CNS. In the CNS, homeostatic balance and modulation of neuroinflammation is mediated by microglia, a type of glial cell that acts as the resident innate immune cells (Tremblay, 2020). Microglia originate from yolk sac primitive macrophages, entering the brain during early embryonic development, around day 9.5 in mice or gestational week 4.5 in humans, which is equivalent to the first trimester (Monier et al., 2007; Ginhoux et al., 2010). Microglia express a variety of morphologies that are closely associated with function. In the healthy brain, surveying microglia are the predominant morphology, using numerous, highly branched, and dynamic thin processes to constantly survey the parenchyma for homeostatic changes (Shigemoto-Mogami et al., 2014; Ueno et al., 2013; Miyamoto et al., 2016; Tremblay, 2021). Surveying microglia have various functions, including modulation of neurons and glial cells, facilitating the formation and pruning of synaptic elements, and maintenance of myelination, and are an integral component of the neurovascular unit (Shigemoto-Mogami et al., 2014; Ueno et al., 2013; Miyamoto et al., 2016; Tremblay, 2021).

**Figure 2:**
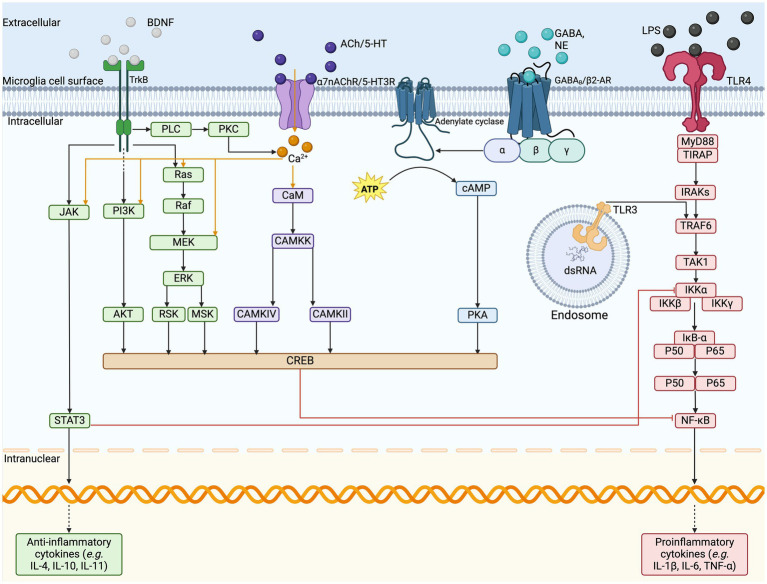
Microglia cell surface sensome and anti-inflammatory pathways. The illustration depicts the cell surface receptors found on microglia and the respective cell signaling cascades. VNS stimulates the release of various neurotransmitters, such as BDNF, ACh, NE, GABA, and glutamate, which act on their respective cell surface receptors on microglia. In response to pro-inflammatory signaling on TLRs, the signal cascades can inhibit the transcription of pro-inflammatory cytokine release and upregulate the release of anti-inflammatory cytokines to resolve inflammation. ACh, acetylcholine; AKT, protein kinase B; ATP, adenosine triphosphate; BDNF, brain-derived neurotrophic factor; CAMKK, calcium/calmodulin-dependent protein kinase; cAMP, cyclic adenosine monophosphate; NE, norepinephrine; dsRNA, double stranded ribonucleic acid; ERK, extracellular signal regulated kinases; GABA, gamma-aminobutyric acid; GPCR, G-protein coupled receptor; IL, interleukin; IRAKs, interleukin-1 receptor associated kinase; JAK, Janus kinase; LCIG, ligand-gated ion channels; LPS, lipopolysaccharide; MEK, mitogen activated protein kinase-kinase; MSK, mitogen and stress activated kinase; MyD88, myeloid differentiation primary response; NF-kB, nuclear factor kappa B; PI3K, phosphoinositide 3-kinase; PKA, protein kinase A; PKC, protein kinase C; PLC, phospholipase C; RSK, ribosomal S6 kinase; RTK, receptor tyrosine kinase; STAT3, signal transducer and activator of transcription 3; TAK1, mitogen-activated protein kinase kinase kinase; TIRAP, TIR domain containing adaptor protein; TLR, Toll-like receptor; TNF, tumor necrosis factor; TRAF6, TNF receptor associated factor 6; VNS, vagus nerve stimulation.

Microglia host a large variety of well-established surface receptors (Figure 2) as part of their sensome, including those for DA, adenosine, opioids, cannabinoids, and CRH (Watters and Pocock, 2014; Liu et al., 2016). The innate immune response in the CNS is initiated by microglial TLRs, NLRs, and triggering receptors expressed on myeloid cells (Rodríguez-Gómez et al., 2020). Stimulation of TLRs leads to NF- κ B and mitogen-activated protein kinase cascades activation, subsequently leading to pro-inflammatory mediator transcription and phagocytosis of nearby damaged neuronal cells (Watters and Pocock, 2014; Rodríguez-Gómez et al., 2020). Microglia also express ARs, including β 1-AR, β 2-AR, α 1A-AR, and α 2A-AR, for NE, as well as α 3, α 5, α 6, α 7, and β 4 nicotinic receptors for ACh, and GABA_A_ and GABA_B_ receptors, all of which promote a neuroprotective, anti-inflammatory microglial phenotype (Watters and Pocock, 2014; Liu et al., 2016). Activation of microglial α 7nAChRs results in transcriptional alterations such as increased antioxidant genes and decreased phosphorylation of NF- κ B, thereby reducing pro-inflammatory cytokine release (Han et al., 2014). Additionally, one study analyzed the role of ACh in LPS-elicited microglial inflammatory response using rat neuron-microglial co-cultures and found that higher levels of ACh reduced the concentration of TNF α and inhibited hippocampal neuronal apoptosis (Li et al., 2019). Inhibition of TNF α by α 7nAChR is mediated by reduced extracellular signal-regulated kinase 1/2 and p38 mitogen-activated protein kinase signaling (Li et al., 2019). Additionally, microglia express the ionotropic glutamate receptors α -amino-3-hydroxy-5-methyl-4-isoxazolepropionic acid (AMPA)-type GluR1-GluR4 and kainate, and all three metabotropic glutamate receptors (Watters and Pocock, 2014; Liu et al., 2016). The AMPA receptors have been shown to both inhibit and stimulate the release of TNF α , whereas metabotropic glutamate and kainate receptors increase TNF*α* (Watters and Pocock, 2014; Liu et al., 2016). There is support for the presence of N-methyl-D-aspartate (NMDA) receptors on microglia, which enhance the release of TNF α , IL-1, and nitric oxide (Watters and Pocock, 2014; Liu et al., 2016).

A neurotransmitter modulator associated with microglial function is BDNF. The neurotrophic factor BDNF plays a critical role in neuronal plasticity and is mainly produced by neurons, where it can be utilized by other neurons or microglia (Andero et al., 2014). Studies have shown that microglia also produce BDNF, although the impact of microglial BDNF is currently debated (Honey et al., 2022; Onodera et al., 2021). Some studies have found expression levels of microglial BDNF to be either absent or too low to noticeably modulate neuronal function (Honey et al., 2022; Onodera et al., 2021). However, others have highlighted the importance of microglia-derived BDNF in learning-induced synapse formation in mice, and its expression on pro-inflammatory microglia following adverse early life experiences (Komori et al., 2024; Parkhurst et al., 2013). Dimerized BDNF binds tropomyosin-related kinase B (TrkB) receptors and p75 neurotrophin receptor in neurons (Wang et al., 2015; Zhang et al., 2003; Frisén et al., 1993). However, the expression of TrkB on microglia also remains a topic of debate due to the heterogeneity of microglia across species, ages, and brain regions (Wang et al., 2015; Zhang et al., 2003; Frisén et al., 1993). One mouse study observed that neuronal BDNF prevented microglia from engulfing mossy fiber synapses in the hippocampus and increased microglial motility and synapse engulfment when BDNF was blocked (Onodera et al., 2021). Another mouse study found that microglial exposure to BDNF reversed LPS-induced inflammatory responses (Charlton et al., 2023). These studies both indicate that BDNF may have some impacts on microglial functions in the hippocampus.

Microglia have high levels of cellular plasticity, resulting in many diverse structural states that allow them to shift between functions of surveillance, neuroprotection and neurotoxicity (Hickman et al., 2013). One phenotype, surveillant microglia, present long processes, capable of crosstalk with neurons and monitoring homeostatic changes in the CNS microenvironment (Savage et al., 2020). Upon homeostatic challenge and across aging, microglia may express states including dystrophic and senescent states (Savage et al., 2020; Bisht et al., 2016). These states often display a more ameboid-like morphology, with shorter, thicker, and less branched processes (Savage et al., 2020; Bisht et al., 2016). Dark microglial states, present in early development and in pathology, display makers of cellular stress (Savage et al., 2020; Bisht et al., 2016). After the detection of an immune insult, microglia can change their morphology, proliferative state, phagocytic activity, and antigen presentation capacity to contribute to an immune response (Bachiller et al., 2018). As part of the pro-inflammatory response and when regulating neuronal activity and homeostasis, microglia can produce pro-inflammatory cytokines and chemokines in the brain, such as IL-6, IL-8, and TNF*α* (Bachiller et al., 2018). Microglial phenotypes are diverse, making them difficult to universally define, as different stimuli and CNS conditions lead to differential responses and states other than the previously defined status “activated” and “resting,” or “M1” and “M2,” being considered limited and not reflecting the current understanding (Watters and Pocock, 2014; Rodríguez-Gómez et al., 2020; Friedman et al., 2018). Indeed, microglia are always active, in health and disease, and they can co-express M1 and M2 markers in their different states (Paolicelli et al., 2022). Microglial release of pro-inflammatory mediators is necessary for physiological processes, acting as a defence and repair mechanism that is held under tight regulation by anti-inflammatory mediators (Rodríguez-Gómez et al., 2020). Previous studies have suggested that microglial involvement in neurodevelopmental disorders, like ASD and schizophrenia, is linked to elevated levels of microglial neuregulin and irregular pro-inflammatory cytokine production associated with altered physiological activities in patients (Ikawa et al., 2017; Rodriguez and Kern, 2011). Some maternal factors, such as immune activation, can also influence prenatal microglial immune reactivity, as shown by a mouse study which demonstrated that microglia could become blunted, presenting a long-lived decrease in immune reactivity, after maternal immune activation (Hayes et al., 2022). *In vitro* this was seen as a reduction of IL-6 and TNF α release from primary microglial cells from the maternal immune activation group after LPS stimulation compared to the control (Hayes et al., 2022). Additionally, *in vivo* results indicated decreased CD68+ lysosomes in microglia, smaller microglia size, and an imbalance in the microglial mitochondrial pathways in the immune activation group compared to the control (Hayes et al., 2022).

## 5 Vagus nerve stimulation

VNS is a neuromodulation technology approved for patients with severe neurological disorders, including drug-refractory epilepsy, stroke neurological sequelae, cluster headaches, and migraines, alongside neuropsychiatric disorders like major depression (Dawson et al., 2021; Fisher et al., 2020; Silberstein et al., 2016). The most common form of VNS is invasive VNS (iVNS), which involves surgical implantation of a programmable pulse generator device for electrical stimulation of the left cervical VN (Howland, 2014). The procedure is typically performed on an outpatient basis under general anesthesia, involving subcutaneous implantation of the generator and attaching the electrode wire to the mid-cervical VN (Howland, 2014). A programmable wand placed outside the skin controls stimulation features, such as the current charge, pulse width, pulse frequency, on/off duty cycle, and more (Howland, 2014). Approved parameters range from 0.25–3 mA for the current intensity, 300–500 μ s for the pulse width, and a frequency of 20–50 Hz, alongside timing parameters (Badran and Austelle, 2022). Since the right VN directly innervates the sinoatrial valve in the heart, stimulation could cause cardiac effects, including bradycardia and asystole (Howland, 2014). Therefore, left VN stimulation has been preferentially approved as the primary treatment modality (Howland, 2014). Adverse effects, such as wound infection and hoarseness, are mostly associated with the surgical procedure and only occur in about 1% of patients (Howland, 2014). Stimulation-related effects are limited to the short period during stimulation and can be mediated by decreasing stimulation parameters, but can include voice alteration, cough, dyspnea, and changes in breathing patterns during sleep, resulting in apneas (Howland, 2014). Due to the invasiveness, the treatment is limited to individuals resistant to conventional therapies, and its high, unsubsidized cost further limits its use (Yap et al., 2020). Regardless, iVNS devices were approved by the United States FDA for refractory epilepsy in 1997 and for chronic treatment-resistant depression in 2005 (Howland, 2014). As of 2021, more than 125,000 patients have received iVNS devices for treatment (Fetzer et al., 2021).

Early studies of VNS in animals found that iVNS had a potent anti-convulsive effect (Zabara, 1992; Lockard et al., 1990). Further studies confirmed the viability of the treatment for epilepsy, as thoroughly outlined in another review (Milby et al., 2009). The iVNS therapy for epilepsy was approved for use in 1997 for adults in the USA and Canada and demonstrates a 50–60% response rate in patients with over 50% seizure reduction (Dolezalova et al., 2022). It also improved the mood of epilepsy patients, leading to further studies into its use in treatment-resistant depression, which was later approved for use in 2005 (Howland, 2014). Other conditions iVNS is approved for include ischemic stroke, tinnitus, traumatic brain injury, and spinal cord injury (Hulsey et al., 2016). Non-invasive VNS devices are being investigated for a wide variety of disorders, such as cluster headaches and migraines, tinnitus, schizophrenia, and ASD (Hasan et al., 2015; Hyvärinen et al., 2015; Nesbitt et al., 2015). Furthermore, the discovery of the inflammatory reflex encouraged new studies to investigate VNS as a possible treatment modality for inflammatory conditions, such as inflammatory bowel disease, rheumatoid arthritis, and Crohn’s disease (Bonaz et al., 2017; Koopman et al., 2016). Clinical studies focusing on iVNS showed efficacy in rheumatoid arthritis and Crohn’s disease in small cohorts, while transcutaneous methods have shown efficacy in alleviating CNS inflammation by altering microglial response to a neuroprotective phenotype in mouse models *in vitro* (Bonaz et al., 2017; Koopman et al., 2016; Zhao et al., 2019). Additionally, both invasive and transcutaneous devices have shown some success in treating inflammatory conditions, like rheumatoid arthritis, by reducing mouse and human serum levels of TNF, IL-6, and IL-1*β* (Hong et al., 2019; Addorisio et al., 2019).

Alternative methods of VNS include transcutaneous VNS (tVNS), which is non-invasive and targets either the ABVN (auricular VNS) or the cervical branch of the VN (cervical VNS) (Howland, 2014). Auricular VNS targets the cymba conchae, which is the only known location with 100% VN innervation (Wang et al., 2021). Stimulation parameters have a much wider range, with current intensity from 0.13–50 mA, pulse width from 20–500 μ s, and frequency between 1–30 Hz (Badran and Austelle, 2022). These parameters can be higher than in iVNS due to the insulative properties of the skin (Badran and Austelle, 2022). Functional magnetic resonance imaging studies have confirmed that auricular VNS stimulates the same brain regions as iVNS, such as the brain stem, hippocampus, amygdala, prefrontal cortex, and thalamus (Badran and Austelle, 2022). Further, the higher stimulation parameters increase brain response without hanging the regional specificity (Badran and Austelle, 2022). One barrier with this method is that the afferent pathway of the ABVN remains poorly understood. FDA-approved devices for auricular VNS include the NEMOS and NET-2000 (Wang et al., 2021). These devices were authorized for the treatment of epilepsy and depression in 2010, and pain management in 2012 (Howland, 2014). Cervical VNS uses gammaCore devices on the anterolateral surface of the neck to target the VN in the carotid sheath (Wang et al., 2021). The electrodes are placed on the sternocleidomastoid muscle, where the VN is close to the surface of the neck and provides a convenient marker for placement (Wang et al., 2023). Stimulation parameters are commonly adjusted up to a maximum of 60 mA, a pulse width of 1,000 μ s, and a frequency of 25 Hz (Miyatsu et al., 2024). This device was approved for the treatment of cluster headaches in 2017, migraine in 2018, and hemicrania continua in 2021 (Howland, 2014). One limitation of cervical VNS concerns the position of the VN. While the stimulation device is placed on the same VN location as iVNS electrodes, the electric pulse must navigate through around 2 mm of skin, 3–6 mm of superficial fascia, and 5–6 mm of the sternocleidomastoid muscle, making selective stimulation difficult and increasing the likelihood of stimulating both afferent and efferent fibers (Yap et al., 2020). However, magnetic resonance imaging (MRI) has shown promise in tailoring stimulations based on individual characteristics, such as skin conductivity and tissue thickness (Kaczmarczyk et al., 2018). It has also been suggested that lower frequency stimulations could favor efferent over afferent fiber activation (Meneses et al., 2016). Both tVNS mechanisms stimulate various brain regions, such as the NTS, parabrachial area, hypothalamus, amygdala, nucleus accumbens, and LC, as shown by MRI (Yakunina et al., 2017). For the purposes of this review, both types of tVNS will be referred to interchangeably in further discussion unless otherwise stated. The value of tVNS as an alternative treatment modality lies in its limited side effects. The common side effects include some pain or itching at the stimulation site, and, in less common cases (<1%), patients may experience nausea or vomiting, headache, heart palpitations, facial drooping, dizziness, and vocal hoarseness (Yap et al., 2020). Despite the possibility of side effects, they are generally less severe and less frequent than those associated with iVNS. Furthermore, tVNS avoids the need for costly surgery, making it a viable option for further research.

## 6 Neuronal mechanisms of vagus nerve stimulation

### 6.1 Microglia and inflammation

One possible action of VNS in the brain is through the modulation of inflammation. Studies testing VNS for rheumatoid arthritis have shown that VNS inhibits joint inflammation and the release of inflammatory cytokines (Zhang et al., 2008). Clinical studies have also indicated that VNS might be beneficial against rheumatoid arthritis owing to a reduction of TNF- α release *ex vivo* and might lead to improvements in disease severity (Koopman et al., 2016; Genovese et al., 2020). In addition, the inflammation that underlies the pathogenesis of CNS diseases, such as multiple sclerosis, Alzheimer’s disease, epilepsy, and schizophrenia, is of great interest for VNS research (Kelly et al., 2022). Experimental studies in animal models have revealed that VNS plays a significant role in altering inflammatory responses (Howland, 2014). Additionally, a human trial in patients with refractory epilepsy found a decrease in IL-8 during long-term VNS (6 months), with no significant changes in the expression of IL-1 β , TNF- α , IL-6 or IL-10 (De Herdt et al., 2009). In a study of cerebral ischemia researchers also observed that VNS downregulated IL-1 β and IL-18 in brain tissues from the cortex, but the neuroprotective effects offered by VNS were reversed by the administration of an α 7nAChR antagonist (Tang et al., 2022). These results support a role of α 7nAChR in the anti-inflammatory action of VNS. Given the anatomical connections of the VN to the NB, the direct modulation of ACh levels may explain the link of this mechanism. A study on rats focused on depression and stress moreover revealed decreased IL-1 β , TNF- α , and IL-6 levels in hippocampal tissues after VNS, as well as morphological changes in hippocampal microglia, notably from amoeboid to surveillant states, with an increased expression of α 7nAChR (Namgung et al., 2022). In traumatic brain injury, VNS reduced TNF- α levels in the serum and brain tissue in a rabbit model (Zhou et al., 2014). Elsewhere, a study showed that VNS significantly reduced the release of pro-inflammatory cytokines such as TNF- α , IL-1 β , and IL-6 in the ischemic penumbra cortex 24-h after ischemia through α 7nAChR activation in microglia from mice subjected to vascular occlusion (Jiang et al., 2014).

Microglia hold a central place in VNS-induced cholinergic anti-inflammatory mechanisms. Numerous CNS conditions have been examined to determine the role of microglia in the actions of VNS, including epilepsy, stress, ischemic stroke and spinal cord injury (Namgung et al., 2022; Chen et al., 2022; Zhang et al., 2021). TLR4 is an important mediator in neuroinflammatory-related disease that is primarily expressed by microglia and has been demonstrated to have a role in traumatic brain injury and ischemic stroke (Yao et al., 2017; Tian et al., 2019). One study on rats found that VNS inhibited the TLR4/myeloid differentiation primary response 88/NF- κ B pathway in microglia, thereby reducing the release of the pro-inflammatory cytokines IL-1 β and IL-6 (Zhang et al., 2021). Activation of α 7nAChR stimulates adenylyl cyclase 6, promoting the degradation of TLR4, which is consistent with their findings of reduced TLR4 expression after VNS in rats (Zhang et al., 2021; Kim et al., 2014; Zhu et al., 2021). Additionally, VNS can increase ACh levels in the rat brain, thereby activating α 7nAChR in microglia after ischemic stroke, which inhibits the peripheral inflammatory response in part through the TLR4/NF- κ B pathway (Zhang et al., 2021; Kim et al., 2014). A study in mice subjected to transient middle cerebral artery occlusion also found that VNS treatment, given for 60 min before, during, and after the occlusion, preserved microglial α 7nAChR expression in the penumbra regions and inhibited NLRP3 inflammasome activation (Xia et al., 2024). Proposed mechanisms for the central reduction of inflammation involve the VNS-induced release of NE from the LC, which has been shown to be essential for the anti-convulsive effects of VNS, by activating β 2-ARs on microglia, astrocytes, and neurons to reduce inflammatory responses via the NF- κ B pathway (Krahl et al., 1998; Laureys et al., 2014). Additionally, activation of the NB triggers the release of ACh to target α 7nAChRs, which induce an anti-inflammatory response (Kaczmarczyk et al., 2018). Studies on spinal cord injury models and stress models in rats found similar results, with VNS downregulating pro-inflammatory cytokine release and promoting microglia to release anti-inflammatory mediators via α 7nAChR upregulation (Namgung et al., 2022; Chen et al., 2022).

Studies have shown evidence of anti-inflammatory effects in human rheumatoid arthritis upon treatment with VNS, as well in reducing the central inflammatory response in murine autoimmune encephalomyelitis (Koopman et al., 2016; Hao et al., 2011). One study, utilizing LPS in mice to induce increased pro-inflammatory cytokine levels found that the whole brain pro-inflammatory cytokine levels were significantly reduced after VNS, alongside a significant reduction in the percent of microglia (CD11b+/CD45^low^) as seen by flow cytometry of whole brains (Meneses et al., 2016). Additionally, they found a decreased expression of the microglia/macrophage marker Iba1 in the hippocampus of VNS-treated mice, suggesting the treatment normalized microglial reactivity which could be linked to reduced CNS inflammation (Meneses et al., 2016). A study also tested VNS in a rat model of LPS-induced demyelination and found a reduced microglial response to inflammation and improved remyelination around the lesion border, indicated by a 57.4% reduction in demyelination compared to the sham (Bachmann et al., 2024). However, there was no preventative effect of VNS on demyelination (Bachmann et al., 2024). They suggest that VNS may enhance microglial clearance of debris to favor remyelination (Bachmann et al., 2024). Additionally, this study found a reduced Iba1 expression and cell count, without a reduction in surveillant microglia, indicating that VNS may reduce microglial reactivity and promote neuroprotective microglial populations (Bachmann et al., 2024). This is supported by a study that used a mouse model of maternal immune activation to study the effect of auricular VNS in the treatment of ASD-phenotypes in offspring (Zhang et al., 2024). Using Iba1 as a microglial marker and CD15, a marker found in immune cells and used as a marker for reactive microglia, they found 7 days of auricular VNS in adult maternal immune activation exposed mice decreased microglial proliferation and decreased the number of reactive microglia in the medial prefrontal cortex (Zhang et al., 2024). They additionally used an anti-IL17a model to compare against VNS treated mice and suggest the mechanism may be mediated through the IL-17a inflammatory pathway (Zhang et al., 2024).

Other hypotheses implicate that the central role of α 7nAChR involves the expression of peroxisome proliferator-activated receptor γ (PPAR γ ), which is upregulated by α 7nAChR activation (Jiang et al., 2015). PPAR γ is a ligand-activated nuclear receptor that plays a role in adipocyte differentiation, lipid metabolism, and insulin resistance, as well as inhibits the synthesis and secretion of pro-inflammatory cytokines in the CNS (Jiang et al., 2015; Kapadia et al., 2008). Hypotheses stipulate that VNS may upregulate the expression of PPAR γ for participation during VNS-induced neuroprotection, while its activation can exert anti-inflammatory effects in both the periphery and CNS (Jiang et al., 2015). In line with this, studies using a rat model of right middle cerebral ischemia have demonstrated that VNS decreased pro-inflammatory cytokine expression and upregulated PPAR γ gene expression via activation of α 7nAChR (Jiang et al., 2015). Additionally, the authors found decreased neuronal damage, and improved neurofunctional recovery after ischemic stroke (Jiang et al., 2015).

The mechanisms underlying the anti-inflammatory effects of VNS occur through various pathways. Another possible pathway is the cholinergic anti-inflammatory pathway, where α 7nAChR in splenic macrophages inhibits the release of pro-inflammatory cytokines (Chen et al., 2018). The connection between the microbiota, gut, and brain, notably through the microbiota-gut-brain axis, can play an important role in modulating inflammatory responses via the VN and is associated with disorders, such as neurodevelopmental, neuropsychiatric and neurodegenerative conditions (Wang et al., 2021). The cells in the gut called enteroendocrine cells detect chemical signals and transmit them to the VN through the enteric neurons, triggering an anti-inflammatory response (Wang et al., 2021). Permeability in the BBB can allow for peripheral immune signaling that triggers an inflammatory response, which can be regulated by the VN. For example, neurodegenerative diseases and ischemic stroke are characterized by functional and structural changes in the BBB (Jin et al., 2023). When glial cells in the brain parenchyma bind to damage-associated molecular patterns, they undergo phenotypic changes leading to an increased release of pro-inflammatory mediators that affect BBB permeability (Jin et al., 2023). Besides, studies have shown that epilepsy is associated with structural and functional changes in the BBB (Kaya et al., 2008; Kaya et al., 2013). VNS treatments were shown to strengthen BBB integrity, likely through the modulation of α 7nAChR’s, notably improving the barrier’s structural and functional components (Kaya et al., 2008; Kaya et al., 2013). Specifically, VNS-mediated α 7nAChR-induced upregulation in splenic macrophages was shown to inhibit the release of pro-inflammatory factors, thereby protecting the initial degradation of the BBB (Chen et al., 2018). Additionally, VNS reversed BBB permeability and decreased pro-inflammatory microglial responses and TNF- α levels in mice with cerebral microinfarction and colitis (Chen et al., 2018). Under this model, the actions of the cholinergic anti-inflammatory pathway provide a possible route to influence CNS inflammation.

### 6.2 Modulation of plasticity: ACh and BDNF

ACh and BDNF are both central neurotransmitters in neuronal plasticity and have been implicated in the mechanism of VNS-mediated plasticity modulation. Activation of the NB was found to be essential to the plasticity-enhancing effects of VNS in the motor cortex (Hulsey et al., 2016). For example, increased ACh release has been associated with increased visual cue detection in the prefrontal cortex and cognitive performance in learning and spatial memory tasks in the hippocampus in rats (Gombkoto et al., 2021). Nicotinic and muscarinic receptors can also modulate the release of other neurotransmitters, such as glutamate, DA, 5-HT, and NE (McGehee et al., 1995; Picciotto et al., 2012). The interaction of the cholinergic system with the dopaminergic pathway has led to suggestions that nAChRs and M1-muscarinic ACh receptors could be involved in dopaminergic dysregulation in patients with schizophrenia (Eickhoff et al., 2022). As previously discussed, ACh also ties into microglial modulation, which could also play important roles in increasing plasticity.

BDNF is another neurotransmitter that plays a crucial role in hippocampal neuronal plasticity. Activation of the TrkB receptor leads to activation of downstream pathways to recruit molecules like phosphoinositide 3-kinase, whose downstream pathways promote cell growth and proliferation (Yang et al., 2020). Studies in rat models have supported these connections through findings indicating that VNS influenced the expression of BDNF, 5-HT, NE, and inflammatory mediators involved in hippocampal neurogenesis (Manta et al., 2009a; Biggio et al., 2009; Follesa et al., 2007). One study observed reduced BDNF mRNA expression in the hippocampal CA1, CA3, and dentate gyrus of vagotomised rats and mice, as well as decreased adult hippocampal cell proliferation and decreased survival of newly born neurons in these animals (Andero et al., 2014; O’Leary et al., 2018). The gut has also been implicated in VN-mediated hippocampal plasticity. The VN facilitates bidirectional communication between the gut and the brain through complexly branching afferent fibers that extensively cover the stomach and receive substantial sensory data (Breit et al., 2018; O’Leary et al., 2018). Evidence from mouse studies support the role of the gut microbiota in influencing hippocampal neuronal plasticity, reducing depression-like behaviors, and altering protein expression in the hippocampus (O’Leary et al., 2018; Bravo et al., 2011). Additionally, the VN processes gut signals, such as mechanical distension and nutrient acquisition, which can, in turn, activate the hippocampus (Min et al., 2011; Wang et al., 2006). One study indicated that gut-derived VN afferent signaling enhanced hippocampus-dependent learning and memory functions in rat models and provided evidence of reduced BDNF in the hippocampus following VN signal loss (Suarez et al., 2018).

The neurotrophic hypothesis of depression and anti-depression action is backed by evidence of decreased BDNF concentrations in mood disorders, with chronic treatment of depression leading to increased BDNF expression (Karege et al., 2005). Early rodent studies found that VNS increased mRNA and protein levels of BDNF in the hippocampus through both acute and chronic stimulation (Biggio et al., 2009; Follesa et al., 2007). Studies using TrkB inhibitors also found that the anxiolytic and anti-depressant-like effects of VNS were inhibited, further suggesting a mechanism involving BDNF/TrkB (Shah et al., 2016). A study using a rat model has shown that chronic and acute VNS increases TrkB receptor phosphorylation in the hippocampus, even at similar TrkB protein levels, and noted increased activation of phospholipase C, gamma 1 and mitogen-activated protein kinase cascades activation/phosphoinositide 3-kinase signal transduction pathways (Furmaga et al., 2012). Treatment with VNS also caused increased phosphorylation of the downstream effector’s extracellular signal-regulated kinase and protein kinase B (Furmaga et al., 2012). Another study on rats found increased BDNF localized in the CA1 and CA2 hippocampal regions, as well as induction of BDNF protein translation after a single VNS session (Olsen et al., 2022). As previously noted, vagotomy, the surgical cutting of the VN, decreased BDNF expression in hippocampal regions, which supports a mechanism for VNS in regulating hippocampal neurogenesis (O’Leary et al., 2018). Overall, these studies strongly support a VNS-induced increase in BDNF/TrkB expression in the hippocampus as a possible mechanism for its anti-depressant action.

### 6.3 The monoamine hypothesis

Monoaminergic neurotransmission of NE from the LC and basolateral amygdala has been hypothesized to be a mediator of VN effects in epilepsy and depression (Landau et al., 2015; Dorr and Debonnel, 2006). The monoamine hypothesis of depression postulates a deficit in NE and 5-HT in the brain, leading to depression (Landau et al., 2015; Moret and Briley, 2011). Therefore, the promotion of NE by VNS is likely associated with the reported antidepressant effects. Pharmacological treatment modalities link 5-HT and NE to the anti-depressant and anti-convulsive effects of VNS (Giorgi et al., 2004; Snead, 1983). Moreover, some recent studies on rodent models have revealed a role for altered serotonergic transmission in the pathogenesis of epilepsy (Bonnycastle et al., 1957; Zhang et al., 2018). The α 2 adrenergic autoreceptors that receive NE are located in the LC pre-synaptically to control the release of NE and are located post-synaptically in projection areas in the cortex to modulate signaling pathways (Shansky and Lipps, 2013). One study utilized positron emission tomography (PET) to measure changes in NE receptor binding in minipigs (Landau et al., 2015). It was observed that VNS reduced the selective α 2-AR antagonist binding potential in limbic, thalamic, and cortical brain regions, which is consistent with NE release (Landau et al., 2015). Furthermore, microdialysis studies in rats exposed to acute VNS demonstrated increased NE in the amygdala, hippocampus, and prefrontal cortex (Follesa et al., 2007; Hassert et al., 2004; Roosevelt et al., 2006). The LC is critically linked to seizure suppression in VNS, indicating the significance of the LC and NE release for the effectiveness of treatment (Krahl et al., 1998). Short exposures to VNS have been shown to activate the NTS, parabrachial nucleus, and LC, while chronic exposure additionally activates the cingulate cortex and DRN (Yuan and Silberstein, 2016). Chronic VNS increased DRN activity to nearly double, associated with increased levels of 5-HT in the brain, while acute VNS had no effect (Dorr and Debonnel, 2006). When the LC was lesioned, VNS did not affect DRN activity, suggesting the importance of the LC as a key mediator between the NTS and DRN for driving the outcome of VNS on 5-HT promotion (Manta et al., 2009b). This mechanism is very promising in explaining the effectiveness of VNS in epilepsy and depression treatments and implicates the modulation of NE and 5-HT release by the VN.

### 6.4 Modulation of ghrelin, oxytocin, GABA, and DA

Additional signaling molecules potentially associated with the VN’s inflammatory response include ghrelin, oxytocin, GABA, and DA. Ghrelin is a growth hormone primarily associated with the stomach, which has receptors expressed on the DMV and can modulate inflammation and protect the BBB (Wang et al., 2021). A study has confirmed that VNS increases ghrelin expression and decreases TNF- α in patients with traumatic brain injury, supporting its role in the central anti-inflammatory response (Bansal et al., 2012).

Oxytocin, released by the posterior pituitary gland, plays an important role in the reproductive systems and exhibits central anti-inflammatory effects through inhibition of pro-inflammatory cytokine release (Wang et al., 2021). The PVN is the primary site of oxytocin production and receives projections from the NTS and projects to the DMV (Wang et al., 2021). Oxytocin is believed to be beneficial for disorders related to social interaction deficit, such as ASD, by activating higher cortical regions and the PVN, and through the rescue of synaptic plasticity in the ventral tegmental area, which is an important region for learning and memory (Sgritta et al., 2019; Hara et al., 2017). Stimulation of the VN increases oxytocin levels and thus may be a mechanism by which VNS exerts its protective effects (Wang et al., 2021).

GABA is an inhibitory neurotransmitter with receptors primarily found in the NTS, DMV, prefrontal cortex, amygdala, and hippocampus (Wang et al., 2021). Studies have found GABA to be important in VNS modulation of working memory, automatic movement inhibition, reduction of cortical excitability, and neuroprotective effects (Wang et al., 2021). Additionally, impairment of GABA_A_-mediated inhibition of neuronal excitability is key to drug-resistant epilepsy (Marrosu et al., 2003; Keute et al., 2021). Application of a single photon emission computed tomography with benzodiazepine receptor inverse agonist, Iomazenil, showed that VNS significantly increased GABA_A_ receptor density and plasticity in human studies (Marrosu et al., 2003; Keute et al., 2021). This suggests that VNS may inhibit the development of epilepsy by reducing neuronal excitability in cortical brain regions associated with epilepsy. One study in rats found that subdiaphragmatic vagal deafferentation resulted in transcriptional changes in the brain associated with schizophrenia and DA alterations in the nucleus accumbens, as well as induced anxiety-like behaviors (Klarer et al., 2014). The behavioral changes were associated with alterations in GABA and NE levels in the limbic system, without functional changes to the HPA (Klarer et al., 2014). These studies support the role of VN afferents in the modulation of GABAergic signaling.

DA is a neurotransmitter and hormone associated with emotion, behavior, and movement, primarily produced by the ventral tegmental area and substantia nigra pars compacta and discharged into the prefrontal cortex and nucleus accumbens (Juárez Olguín et al., 2016). The formation of DA from L-DOPA is catalyzed by DOPA decarboxylase, but DA is also a precursor to NE through degradation by dopamine- β -hydroxylase in the presence of L-ascorbic acid and molecular oxygen (Juárez Olguín et al., 2016). Studies have shown that stimulation of the right VN neurons of the nodose ganglion activated the ventral tegmental area and substantia nigra pars compacta and were sufficient to induce DA release, implicating a role for the VN in DA manipulation (Han et al., 2018). Another study confirmed that stimulation of the VN at the cervical site stimulated the ventral tegmental area and substantia nigra pars compacta, but left VN stimulation did not activate these regions (Brougher et al., 2021). Alternatively, the VN may stimulate the production of DA to the prefrontal cortex through stimulation of the LC (Devoto et al., 2005). The consensus of DA manipulation by VNS is not well established, and future studies are warranted to establish a strong connection.

## 7 Promising effects of vagus nerve stimulation on neurodevelopmental disorders

### 7.1 Pediatric epilepsy

Epilepsy is a neurodevelopmental disorder characterized by chronic, uncontrolled seizures affecting over 65 million people worldwide, with 10.5 million being children (Moshé et al., 2015; Guerrini, 2006). Chronic seizures are especially harmful to children, as they interfere with physical and mental growth and can lead to psychiatric comorbidities like depression, anxiety, and ADHD (Ji et al., 2019). When VNS was initially approved as a treatment for epilepsy in 1997, it was for refractory epilepsy in patients aged 12 years or older who failed other therapeutic approaches (Ji et al., 2019). However, in 2017, the common VNS system was approved for patients aged 4 years or older who exhibit epilepsy refractory to antiepileptic medication (Ji et al., 2019). This made the therapy more available to afflicted individuals and allowed researchers to study the effects of VNS in children as a possible treatment for other childhood neurodevelopmental disorders.

Treatment for epilepsy is based on many factors. Currently, there are over 40 antiseizure medications available that offer a wide variety of benefits and drawbacks, taking into account different lifestyle and condition characteristics (National Institute of Neurological Disorders and Stroke, 2024). However, one-third of epilepsy patients do not respond to antiseizure medications and are at higher risk of mortality (Ji et al., 2019; National Institute of Neurological Disorders and Stroke, 2024). This leads to considering other options, such as dietary changes, surgery, or stimulation devices (Ji et al., 2019; National Institute of Neurological Disorders and Stroke, 2024). A ketogenic diet reduces carbohydrate intake to allow the body to rely primarily on fats for fuel and has been particularly effective in reducing seizures in children (National Institute of Neurological Disorders and Stroke, 2024). However, it is a self-regulated diet that can be very challenging for patients to maintain long-term (National Institute of Neurological Disorders and Stroke, 2024). Surgery is a more extreme treatment for those who are unresponsive to other options and can be very risky and sometimes ineffective (National Institute of Neurological Disorders and Stroke, 2024). Alternatively, neurostimulation treatments are available for patients who do not respond to other treatment options and are categorized into three models: iVNS, responsive stimulation, and deep-brain stimulation (National Institute of Neurological Disorders and Stroke, 2024). VNS has been shown to have anticonvulsive effects as well as positive effects on mood, behavior, and cognition in patients with epilepsy (Aalbers et al., 2012). However, while VNS is an effective treatment, the underlying mechanisms are not fully understood, making it difficult to gauge who will be a responder and best benefit from the treatment (Table 1).

**Table 1:** VNS therapeutic effects for inflammation and neurodevelopmental disorders: preclinical and clinical findings.

Disease condition	Authors	Study design	Main findings	Caveats and limitations
Inflammation (preclinical)	Murray et al. (2021)	Utilized adult 8–10 week, C57BL/6 mice, wild type mice and genetically altered mice, not expressing ChAT. Mice were exposed to 20 min of VNS or 20 min of sham stimulation. Stimulation of anesthetized mice exposing right cervical VN, using a bipolar hook electrode, the VN was transected to allow stimulation of either (a) efferent VN, (b) afferent VN (c) VN left intact. *In vitro* induction of LPS 10 min post-stimulation and at 1-h post-stimulation. Five minutes before sham stimulation or VNS, injecting anesthetized mice with selective β2-adrenergic receptor antagonist, or PBS. Quantitative PCR was conducted to evaluate RNA from spleen, MLN, inguinal lymph node. ELISA kit was used to determine serum concentrations of TNF-α.	Serum TNF-α levels were significantly reduced 1 h post LPS induction in mice exposed to VNS (afferent, efferent or VN left intact).	As a preclinical model it shows some validation for mechanisms but lacks direct translatability since they do not use approved devices nor approved methods for humans.
Inflammation (preclinical)	Bachmann et al. (2024)	Use of MS rodent model in 46 Lewis rats. Demyelination and secondary inflammation were induced through the injection of LPC in the corpus callosum. The rats were divided into two groups, differing with regards to the duration of VNS stimulation. The rats in both groups received either cVNS, exposure to VNS for 1 min, or sham VNS. The effect of VNS at 3 days post LPC injection, when the lesion is present, and levels of inflammation are peaking, was compared to the effect of VNS at 11 days post injection, when fibers are partly remyelinated. During the last time point of VNS stimulation of group one the induced lesion was still demyelinated, and inflammation levels were at peak level. For group 2, during the last time point of VNS stimulation the lesion was at a partially remyelinated state. Proteomics and immunohistochemistry analysis were performed.	cVNS was shown to have a positive effect on microglial and astrocytic reactivity, while the effect of 1 min VNS appeared to be small. At 11 days post-injection, cVNS increased remyelination at a rate 57.4%, compared to sham stimulation. At the timepoint of completing lesioning, cVNS did not appear to have a beneficial effect regarding the level of demyelination after injury of the corpus callosum. Immunohistochemistry analysis revealed, a reduction of microglial and astrocytic reactivity in the cVNS exposed group at the time point of assessment 3 days post injection with the demyelinating agent LPC within the lesion and in the lesion borders. cVNS further lowered the levels of Olig2+ cells at 3 days point injection and increased levels of remyelination at 11 post LPC injection. cVNS was further associated with the activation of glutamatergic synaptic pathways.	The model only looks at the response in females which may limit its translatability as differences in male response were not analyzed,
ASD (preclinical)	Zhang et al. (2024)	CD57BL/6 pregnant mice received i.p. injection of either 20 mg/kg of poly (I:C) or saline at E12.5. The 6-week-old pups were divided into two groups to receive, under anesthesia, either VNS or control stimulation. In the VNS group electrodes were placed in the innervation area of the upper and lower ABVN. In the control group, electrodes were placed outside the innervation area. Stimulation occurred for 30 min daily, 7 days in a row. At 6 weeks after birth, pups whose mothers were exposed to saline were injected with sterile cerebrospinal fluid (sham group), pups whose mothers were exposed to Poly (I:C) were injected with either sterile cerebrospinal fluid (ASD group), or with an IL-17a antibody (anti IL-17a group). Behavioral assessments included open field test and three chamber social preference test at 6 weeks after birth. Western blot test, and ELISA were applied to perform proteomics analysis.	Only taVNS at high intensities reduced social disruptions and anxiety-like behavior. Heightened levels of IL-17a were found in the mPFC of ASD mice. Levels of IL-17a were reduced through taVNS. Increased density of Iba1+ microglia were detected in the ASD group, compared to controls. In the ASD group, a higher number of CD16 expression microglia was found. In the taVNS stimulated mice, a reduced number of CD16 expressing microglia was found. Blocking of IL-17a receptors in the mPFC of ASD mice lowered the overall microglia count, and reactive, CD16 expressing microglia. Blocking of IL-17a receptor further improved social disruptions in ASD mouse model.	Morphological states of microglia are multi-dimensional, division of microglial morphology and functioning into two distinct states is too simplistic.
Schizophrenia (preclinical)	Klarer et al. (2018)	Male rats underwent a complete removal of all afferent fibers to induce a model of SDA. Use of next generation mRNA sequencing to investigate genome-wide transcriptional changes, in NAc and PFC, in SDA group, compared to controls. Behavioral testing in domains implicated in schizophrenia-like behavior (sensorimotor gating, selective attentional learning, amphetamine sensitivity). Assessments of dopamine levels, and levels of dopamine metabolites in PFC and NAc were conducted.	Increased levels of dopamine and dopamine metabolites in NAc. SDA disrupted sensorimotor gating, and attentive control required for associative learning. SDA induces transcriptional alterations regarding functional networks implicated in schizophrenia. More transcriptional changes were observed in NAc. Transcriptional changes in NAc were corresponding more to those characteristic of schizophrenia then those observed in the PFC.	Sham group, while being exposed to surgery, did not receive any invasive physiological manipulations; SDA model leaves half of the vagal efferents intact; CCK test to assess completeness of SDA procedure; Use of male rats only; No animal model of schizophrenia was used.
Schizophrenia (clinical)	Hasan et al. (2015)	20 patients with schizophrenia were included, in a randomized, controlled, double-blind study. The patients were assigned to either an active tVNS or a sham tVNS group. The active tVNS condition consisted of daily active stimulations of the left auricle for 20 weeks. The sham tVNS condition consisted of daily sham stimulations for 12 weeks, and 14 weeks of consecutive active tVNS. Using a PANNS scale, positive and negative symptomatology was assessed at baseline, and after the interventions. Symptoms of depression, neurocognitive functioning and safety parameters were assessed. Further the presence of side effects was monitored, and the levels of pro-inflammatory cytokines at baseline and after intervention were assessed.	No effects on PANNS score reflecting change in positive or negative symptomatology. No significant differences between groups in terms of measures of neurocognitive functioning or safety.	Small sample size; low compliance rate, as patients frequently did not apply the stimulation device as instructed. Intensity of tVNS stimulation not standardized. Differences in terms of severity of psychopathology between tVNS and sham group. Patients at baseline displayed only mild symptomatology.
Epilepsy (clinical)	Suller Marti et al. (2020)	46 Patients diagnosed with generalized epilepsy, either Lennox–Gastaut syndrome or genetic generalized epilepsy were included. The patients had VNS device implanted between 1997 and 2018. Treatment response, as well as occurrences of complications were assessed.	Overall seizure reduction of 50% or more in 41.7% of Lennox–Gastaut syndrome patients, and 64.7% of genetic generalized epilepsy patients was detected. Highest reduction in generalized tonic–clonic seizures. Hospital admissions were less frequent after implantation, with 91.3% before implantation of the device, to 43.5% after the implantation.	Inclusion of large age range (17.8–31 years); Average age significantly differed between the two groups. Strong variability regarding the length of time that has passed since implantation of the device.

ASD, autism spectrum disorder; CCK, cholecystokinin; CD16, clusters of differentiation; ChAT, choline acetyltransferase; cVNS, continuously cycled vagal nerve stimulation; ELISA, enzyme linked immunosorbent assay; IL-17a, interleukin-17A; i.p., intraperitoneal; LPC, lysolecithin; LPS, lipopolysaccharide; MLN, mesenteric lymph nodes; mPFC, medial prefrontal cortex; mRNA, messenger ribonucleic acid; MS, multiple sclerosis; NAc, nucleus accumbens; PANNS, Positive and Negative Syndrome Scale; PBS, phosphate buffered saline; PCR, polymerase chain reaction; PFC, prefrontal cortex; poly (I:C), polyinosinic:polycytidylic acid; RNA, Ribonucleic acid; SDA, subdiagraphic vagal deafferentiation; taVNS, transcutaneous auricular vagus nerve stimulation; TNF alpha, tumor necrosis factor alpha; tVNS, transcutaneous vagus nerve stimulation; VN, vagus nerve; VNS, vagus nerve stimulation.

Seizures are defined as the manifestation of abnormal, excessive, and hypersynchronous discharges of cortical neurons (Bromfield et al., 2006). Epilepsy is a chronic CNS disorder characterized by recurrent and unprovoked seizures (Bromfield et al., 2006). Excessive electrical activity in neurons causes involuntary movements, sensations, behaviors, and emotions, as well as loss of awareness, with lingering effects lasting up to hours (National Institute of Neurological Disorders and Stroke, 2024). After-effects can include drowsiness, weakness, and confusion (National Institute of Neurological Disorders and Stroke, 2024). The initiation of a seizure is identified by concurrent high-frequency bursts of action potentials and hyper-synchronization of a neuronal population, causing sustained depolarization (Bromfield et al., 2006). Epileptic seizure severity is believed to be correlated to neurotransmitter and central inflammatory functions, such as the overstimulation of glutamatergic NMDA receptors by tryptophan metabolites, notably kynurenine, which is implicated in seizure generation (Majoie et al., 2011). The cause of epilepsy is unknown, but genetic factors, developmental brain abnormalities, infection, or trauma have been implicated (National Institute of Neurological Disorders and Stroke, 2024). Genetically induced epilepsy has been linked to hereditary and sporadic causes, with at least 500 identified genes estimated to be involved (National Institute of Neurological Disorders and Stroke, 2024; Hajtovic et al., 2022). For example, children with Lennox–Gastaut syndrome, Dravet syndrome, and Tuberous Sclerosis Complex (TSC) can be at risk for epileptic seizures (National Institute of Neurological Disorders and Stroke, 2024). Several genes implicated in epilepsy encode ion channels, and carboxylase or are associated with neuronal migration (National Institute of Neurological Disorders and Stroke, 2024; Hajtovic et al., 2022).

Proposed mechanisms for the action of VNS involve desynchronization of neuronal activity, hippocampal plasticity, anti-inflammation, and neurotransmitter modulation (the monoamine hypothesis), as previously mentioned in this review. In addition to these mechanisms, it is believed that upward activity induced by VNS affects the limbic system (e.g., hippocampus, amygdala, hypothalamus) and cortex, suppressing epileptic waves and cerebral blood flow (Fukuda et al., 2024). VNS is also believed to change GABA and NE nerve activity and amino acid metabolism, increasing GABA in the piriform cortex (Fukuda et al., 2024). Additionally, anti-inflammatory effects, such as reduced neurotoxin concentrations, normalized cortisol levels, and the regulation of cerebral blood flow allow to reduce seizures (Fukuda et al., 2024). Studies have also shown that VNS results in changes in tryptophan concentrations in the CNS, which would decrease the stimulation of NMDA receptors (Majoie et al., 2011). Overall, it is believed that VNS acts through multiple mechanisms to decrease seizure activity in patients.

In addition to these cellular mechanisms, VNS is also affected by genetic mutations linked to epilepsy. As a highly heterogeneous condition, the efficacy of VNS is variable, with different response rates based on the patient’s genetic mutations. A meta-analysis determined that pediatric patients with TSC exhibit a 68% rate of ≥50% seizure reduction. In comparison, patients with Dravet syndrome had a ≥ 50% seizure reduction rate in 41% of cases, with no patients achieving complete seizure-freedom (Hajtovic et al., 2022). Patients with Rett syndrome displayed an 86% response to VNS among 7 patients, while another meta-analysis of 11 patients showed that 82% of patients had a ≥ 50% reduction in seizure frequency (Hajtovic et al., 2022; Xie et al., 2022). In a study on Lennox–Gastaut syndrome, a pair of female monozygotic twins received VNS, with one experiencing a 60% reduction in seizure frequency while the other had no reduction (Xie et al., 2022). This suggests that the effectiveness of VNS may be influenced by environmental (including prenatal) factors in addition to genetic predisposition. However, further studies are required to validate this finding. Studies have also shown that the response to VNS tends to improve over time with chronic usage (Starnes et al., 2019). In addition, studies focusing on children have highlighted the importance of age in determining VNS efficacy, suggesting younger children tend to demonstrate more significant improvements (Ji et al., 2019). This paper provides a comprehensive review of previous data on the efficacy of VNS in children (Ji et al., 2019).

Overall, VNS has proved its efficacy as a treatment modality for children and adults with refractory epilepsy. Future researchers should attempt to determine the mechanisms behind VNS’s therapeutic actions and analyze the role of genetic and environmental influences. This data could help determine markers for responders that could increase the specificity of its use. A review on the use of tVNS for epilepsy determined the need for a common protocol among studies to avoid underestimation of anti-convulsive effects, reporting a weekly seizure reduction variance of 12.5–50%, a monthly seizure reduction of 8.3–64.5%, and a study timeline-based reduction of 30–65% (Lampros et al., 2021). Additionally, the studies reviewed supported increased efficacy in chronic use, with one study reporting a 23% increase in seizure reduction from weeks 8–16, with diminishing increases after week 16 (Lampros et al., 2021). Of the studies analyzed, three reported no differences in response to tVNS between age or gender (Lampros et al., 2021). Regarding adverse effects of tVNS, common side effects included headaches, and ear pain in around 15–20% of patients, as well as skin irritation and rashes (Lampros et al., 2021). Other common symptoms include nasopharyngitis, gastrointestinal symptoms such as nausea and diarrhea, fatigue, and dizziness (Lampros et al., 2021). One study analyzed transcutaneous auricular VNS using the NEMOS device in epilepsy patients but could not draw conclusive results due to limitations related to the device’s usability (Sabers et al., 2021). While non-invasive VNS can be more accessible, it appeared to be limiting in its need for active participation by the patients and possible lifestyle disruptions, as the study required four hours to use the device daily, and issues with electrode connection exacerbated frustrations (Sabers et al., 2021). As tVNS is still a developing treatment option, it shows promise with efficacy and tolerability. Standard protocols are required to continue the advancement of studies and options to make the device more lifestyle friendly for epilepsy patients would be beneficial in broadening its usability.

### 7.2 Schizophrenia

Schizophrenia is a chronic psychiatric disorder represented by positive (e.g., delusions, hallucinations), negative (e.g., social withdrawal, apathy), and cognitive (e.g., learning and memory impairments) symptoms (DSM Library, 2024). According to the World Health Organization, about 24 million people, approximately 1% of the world’s population, are affected by schizophrenia globally, ranking the disease as a serious cause of disability worldwide (Li et al., 2023). Unfortunately, effective treatments for schizophrenia have remained limited, leading to increased disability due to comorbidities, chronic behavioral changes, and increased suicide rates (Laursen et al., 2012). Diagnosis of schizophrenia utilizes the identification of psychosis as a first step but requires additional factors, such as chronicity, dysfunction, and presence of depression or mania to differentiate it from other psychotic disorders (van Os and Kapur, 2009). Studies have determined that prenatal factors, such as maternal infection, stress, or malnutrition, contribute to some cases of schizophrenia (van Os and Kapur, 2009; Ben-Azu et al., 2022).

Pharmacological treatments for schizophrenia are designed to antagonize DA receptors (van Os and Kapur, 2009). The Texas Medication Algorithm Project has developed a six-stage pharmacotherapeutic algorithm for schizophrenia treatment that utilizes first-and second-generation antipsychotics, as well as electroconvulsive therapy (Haddad and Correll, 2018). However, these treatments are often associated with risks of motor, reproductive, and metabolic disorders, such as agranulocytosis, extrapyramidal symptoms, obesity, diabetes, and reproductive dysfunction (Haddad and Correll, 2018; Obukohwo et al., 2024). The specific mechanism behind antipsychotic drugs remains unclear, but they are believed to fall into three categories: high DA antagonism and low 5-HT inhibition, moderate-to-high DA antagonism and high 5-HT blockade, and low DA antagonism and high 5-HT antagonism (Haddad and Correll, 2018). The specific risks of weight gain, reproductive impairment, or extrapyramidal symptoms depend on the specific antipsychotic drugs involved, although they all convey low to high risk in one of these categories (Haddad and Correll, 2018). The response rate of antipsychotics is low, with the most effective treatment having a response rate of 33% after three months, but also being associated with severe side effects (Haddad and Correll, 2018). Also, antipsychotics are only clinically effective for positive symptoms, with unsatisfactory results for negative symptoms and cognitive symptoms (Haddad and Correll, 2018). This lack of effective and safe treatment options has led to the development of alternative therapies targeting the inflammatory pathways involved in the pathogenesis of schizophrenia, such as VNS.

The pathophysiology of schizophrenia is multifaceted and includes, but is not limited to, decreased gray matter, enlarged ventricles, and focal alteration of white matter tracts in patients (van Os and Kapur, 2009). The DA hypothesis of schizophrenia posits an increase in DA in the brain linked to the schizophrenia phenotypes, supported by studies finding increased DA synthesis and release in mesolimbic brain region (e.g., striatum) with decreased levels in mesocortical area (e.g., prefrontal cortex), supported by preclinical and clinical studies (van Os and Kapur, 2009; Ben-Azu et al., 2018). Functional MRI analyses have shown abnormal network response to DA, with areas of hyper-and hypoactivity across different brain regions (van Os and Kapur, 2009). The causes of disturbances in dopaminergic neurotransmission in schizophrenia have been unclear. However, inflammation has been considered a possible mechanism behind the neurochemical alteration (Müller and Bechter, 2013). The inflammatory cytokine IL-1 β , which converts rat mesencephalic progenitor cells to a dopaminergic phenotype, and IL-6, which decreases serotonergic neuron survival in the fetal brain, both influence the development of neurotransmitter systems, especially those involved in schizophrenia (Müller and Bechter, 2013). Rodent models of maternal immune activation support this, as they have been shown to contribute to increased mesencephalic dopaminergic neurons in the fetus, likely associated with the increased DA production seen in schizophrenia (Müller and Bechter, 2013). These maternal immune activation models and studies of human cases are linked to an increased risk of schizophrenia associated with respiratory and reproductive tract infections, specifically those linked to increases in maternal IL-8 (Müller and Bechter, 2013).

A different hypothesis linked to inflammatory responses is the microglial hypothesis of schizophrenia, which suggests that the release of pro-inflammatory cytokines, kynurenines, nitric oxide, and reactive oxygen species by microglia leads to neuronal degradation and white matter abnormalities (Busse et al., 2012). Studies on humans supporting this hypothesis have found increased levels of peripheral benzodiazepine receptor, notably expressed by reactive microglia, and HLA-DR+ microglia, a major histocompatibility complex (MHC) class II marker that may be linked to antigen presentation, in acute psychotic episodes early in the disease course, but another paper found this was not the case in patients with chronic schizophrenia, pointing to certain immune alterations occurring as a function of the disease course (Busse et al., 2012). One study found an increased density of HLA-DR+ microglia in the hippocampus in patients with positive symptoms (paranoid schizophrenia), which may be linked to increased IL-1 β and IL-2 secretion as well as complement-dependent pathways that regulate DA-related brain signaling to modulate monoaminergic neurotransmission (Busse et al., 2012). The impairment of microglial functions in schizophrenia pathology is supported by a variety of evidence, including behavioral and molecular enrichments, as well as post-mortem transcriptomic studies of patients (Zhuo et al., 2023; Ormel et al., 2020; Gober et al., 2022; Nguyen et al., 2023). Genetic studies have shown increased expression of well-known microglial genes, notably TREM2, APOE, CX3CR1, IRF8, P2RX7, and P2RY12 in schizophrenia patient-derived microglial cells (Ormel et al., 2020; Koskuvi et al., 2024). Additionally, increased expressions of IBA1, P2RY12, MHC class II, CD16, CD32a, CD64, and CD68 have been detected, which are considered motility and phagocytic markers of microglia, often associated with increased pro-inflammatory actions (Ormel et al., 2020; Koskuvi et al., 2024). However, it is worth noting that the expression of these markers may depend on age, brain region, and sex (Gober et al., 2022; De Picker et al., 2021).

Another mechanism of schizophrenia may be the lack of glutamatergic neurotransmission caused by NMDA receptor hypofunctionality, as well as disinhibition of GABAergic system and serotonergic impairments (Müller and Bechter, 2013). The glutamatergic hypothesis of schizophrenia is supported by studies showing altered NMDA receptor expression levels in patients with schizophrenia, and the mimicking of psychotic-like behaviors in healthy subjects and experimental animals given NMDA receptor antagonists (Espana et al., 2021; Ben-Azu et al., 2023). The body’s endogenous NMDA antagonist, kynurenic acid, is produced during inflammatory immune responses when the enzyme indoleamine 2,3-dioxygenase gets inhibited, and acts as a natural mediator of NMDA receptor activity (Müller and Bechter, 2013). Additionally, a recent study found that NMDA receptor surface trafficking was altered in patients with schizophrenia (Espana et al., 2021).

So far, there have been few studies exploring VNS as a treatment modality for schizophrenia. One study using tVNS found no differences in schizophrenia symptoms between tVNS-treated and sham groups over a 26-week trial (Hasan et al., 2015). However, the results remained inconclusive due to poor patient adherence to the treatment protocol (Hasan et al., 2015). Another study in rodent models found that chronic VNS can effectively reverse hyperactivity of the hippocampal and ventral tegmental area, mesolimbic dopaminergic dysfunction, and positive schizophrenia-associated symptoms (Perez et al., 2014). Furthermore, studies combining VNS with neuroimaging techniques observed that VNS decreases hippocampal activity, possibly through increased GABAergic signaling (Capone et al., 2015; Smucny et al., 2015). Moreover, improved cognition in domains responsible for attention, memory consolidation, and executive function has been linked to VNS in patients with epilepsy, Alzheimer’s disease, and depression (Smucny et al., 2015). The mechanisms of VNS that are relevant to schizophrenia include increases in NE, ACh, and 5-HT, as well as the manipulation of GABAergic signaling (Smucny et al., 2015). α 7nAChRs are believed to have reduced expression in patients with schizophrenia, leading to hippocampal hyperactivity (Smucny et al., 2015). Therefore, the increased release of ACh by VNS could stabilize this function (Smucny et al., 2015). Similarly, increases in 5-HT may also decrease hippocampal hyperactivity by modulating GABAergic signaling through the 5-HT2, 3, and 4 receptors (Smucny et al., 2015). Activation of the 5-HT1 receptor has been associated with hippocampal neurogenesis, acting as a possible mechanism to restore pattern separation deficits, while 5-HT3 receptors could relieve inhibition of ACh release (Smucny et al., 2015).

The modulation of the immune system may be another way by which VNS affects schizophrenia. Previous studies have shown that the expression of α 7nAChR is reduced in patients with schizophrenia and animal models of schizophrenia (Corsi-Zuelli et al., 2017; Freedman et al., 1995; Leonard et al., 2000). α 7nAChR plays a role in NMDA and GABA receptor activity. Altered α 7nAChR activity has been linked to altered NMDA/GABA receptors and related neuronal functions (Corsi-Zuelli et al., 2017). Of note, kynurenic acid, a popular NMDA receptor inhibitor and a non-competitive α 7nAChR-antagonist, is associated with decreased glutamatergic and cholinergic neurotransmission (Corsi-Zuelli et al., 2017). Different studies have shown that targeting α 7nAChR could lead to beneficial outcomes for schizophrenia patients. A study has shown that the administration of nicotine reversed hypofrontality, a state of decreased cerebral blood flow in the prefrontal cortex, through inhibitory neurons in experimental animal models of addiction and schizophrenia (Koukouli et al., 2017). Another study used a nicotinic agonist known to modulate α 7nAChR and they found significant improvements in the negative symptoms in patients with schizophrenia (Freedman et al., 2008). Indeed, it is estimated that around 70% of people with schizophrenia smoke cigarettes, a fact that is believed to be unknowingly associated with the benefits of α 7nAChR activation (Tregellas and Wylie, 2018). However, since nicotine has side effects such as anxiety, nausea, and mood changes, and is difficult to turn into therapy, VNS may be a feasible alternative to target α 7nAChRs, being well tolerated (Tregellas and Wylie, 2018).

### 7.3 Autism spectrum disorder

ASD is a neurodevelopmental disorder best characterized by poor social interaction and communication, limited interests, and stereotypical behavior (Shivaswamy et al., 2022). Other impairments can include increased anxiety and fear responses, leading to behaviors such as tantrums, aggression, and phobias (Shivaswamy et al., 2022). It affects approximately 1 in 36 children (around 2% of the population), with an increasing prevalence (Shivaswamy et al., 2022; Jin and Kong, 2017). ASD is four times more prevalent in males, as shown by an earlier age of onset and poor response to treatment compared to females, suggesting a sex-related difference (Marotta et al., 2020). Cognitive behavioral therapies are an effective approach for treating anxiety in individuals with ASD and depend on extinction learning for the treatment of ASD-related phobias (Shivaswamy et al., 2022). Intensive behavioral therapies can promote adaptive plasticity and can lead to significant improvements in intelligence, communication, and social skills in individuals with ASD, but are also very time-consuming and expensive (Engineer et al., 2017). Furthermore, a significant number of children do not respond to these therapies, thereby limiting the treatment’s usefulness (Engineer et al., 2017).

Neuroimaging techniques have found abnormalities in the grey and white matter with regional differences in individuals with ASD compared to their typically developing counterparts (Ha et al., 2015). Total brain volume in children with ASD was also determined to be accelerated at around 2–4 years of age, with enlargement of the frontal and temporal lobes, but it tends to be decreased or present no difference in older individuals (Ha et al., 2015). Additionally, studies have identified an accelerated expansion of the cortical surface area, but not thickness, before 2 years of age, which may be associated with the impaired maturation of cortical white matter (Ha et al., 2015). Numerous studies have made a clear connection between the development of ASD and genetic factors, as siblings have a 100-fold increased risk compared to the general population, and monozygotic twins have a 36 to 91% concordance rate, although this does not take into account the effects of the shared maternal and postnatal environment (Marotta et al., 2020).

Alterations in GABAergic and glutamatergic systems are believed to be potential pathological mechanisms for ASD (Marotta et al., 2020). Magnetic resonance spectroscopy has shown reduced glutamate concentrations in the striatum of adults with ASD and reduced GABA levels in the motor, visual, auditory, and somatosensory areas (Marotta et al., 2020). Furthermore, plasma levels of GABA and glutamate are altered in children diagnosed with ASD, leading to an imbalance in excitatory and inhibitory mechanisms (Marotta et al., 2020). Glutamate and its NMDA and AMPA receptors have also been implicated in ASD (Marotta et al., 2020). Rodent models have shown overexpression of NMDA receptor subunits, leading to enhanced synaptic currents and amplified postsynaptic plasticity (Marotta et al., 2020). Meanwhile, AMPA receptors displayed significant decreases in their GluA2 subunit in the hippocampus (Marotta et al., 2020). 5-HT is another neurotransmitter that has been implicated in the development of ASD during early neonatal brain development (Marotta et al., 2020). Studies have shown higher levels of 5-HT transporter or 5-HT in children with ASD and in animal models, while post-mortem studies showed reductions in 5-HT receptor binding (Marotta et al., 2020). Additionally, children with ASD were found to have significantly lower levels of serotonin at the ages of two and five, with a slight increase with age, while healthy children showed elevated 5-HT at this age, with a decline after puberty (Marotta et al., 2020). It is hypothesized that dysfunctional DA level plays a role in the development of ASD, as individuals with ASD demonstrate reduced DA release in the prefrontal cortex and reduced neural response in the nucleus accumbens (Marotta et al., 2020). Some other neurochemicals implicated in the development of ASD include ACh, N-acetyl aspartate, oxytocin, and melatonin (Marotta et al., 2020).

VNS has been proposed as a treatment for ASD due to the unpredictable response to medication and the potential limitations of behavioral therapies (Black et al., 2023). One study investigated the use of auricular tVNS in patients with ASD and found that it was well-tolerated, with high completion rates, tolerability, and delivery of intended stimulation (Black et al., 2023). The study also observed an improvement in anxiety and sleep scores in a small cohort of children after a 2-week period (Black et al., 2023). Another study indicated that VNS improved the recognition of emotion and salient social cues, which are commonly impaired in individuals with ASD (Colzato et al., 2017). However, a separate study involving a small group of patients (n = 7) with both refractory epilepsy and ASD found no significant effects on quality of life, cognitive abilities, or reduction in seizure frequency two years after undergoing iVNS implantation (Danielsson et al., 2008). This highlights the need for further research on the effectiveness of VNS, especially in patients with multiple health conditions. Since this study was published, other studies have tested VNS in children with ASD and have yielded promising results. One study included children with both epilepsy and ASD and found that two of four children who achieved seizure control also had changes in their autism diagnosis, from autism to suspected autism (Wang et al., 2022). This study also observed a significant reduction in autistic behaviors related to language, social skills, and self-help, indicating the possible effects of VNS on neurocognitive function (Wang et al., 2022). However, it is important to consider the potential influence of a child’s age and previous interventions on these results (Wang et al., 2022).

The mechanisms of VNS that may support its usage in ASD involve the modulation of the glutamatergic system, 5-HT, and DA. One study found that VNS activated NDMA receptor-and AMPA receptor-mediated TrkB in the NTS, without changes in BDNF concentration (Liu et al., 2024). Therefore, VNS may be able to modulate glutamate concentrations through the NTS in the CNS. Furthermore, studies support the modulation of 5-HT by chronic VNS (Liu et al., 2024). Children exposed to VNS may be able to express normalized levels of 5-HT through the critical developmental period (Liu et al., 2024). The role of the VN in DA production is less well established, but studies support that the VN can stimulate DA production in the ventral tegmental area and substantia nigra pars compacta or through the LC, which project to the prefrontal cortex (Liu et al., 2024). Overall, while further studies are needed for clarification, these studies support the notion that VNS may have positive effects on mood regulation and could potentially be a promising therapy for ASD.

### 7.4 Attention-deficit/hyperactivity disorder (ADHD)

ADHD is a neurodevelopmental disorder characterized by hyperactivity, impulsivity, and attentional difficulties (Zaehle and Krauel, 2021). Most individuals show symptoms in childhood, with a prevalence of about 5% in school-aged children ( ≥ 18 years old) worldwide, and 2.5% in adults (Cortese et al., 2018). Memory performance, encoding, and consolidation can be affected, and the ability to adjust behavior to situations and anticipate outcomes (Zaehle and Krauel, 2021). While not associated with increased mortality, ADHD in children can accumulate additional financial costs, with studies finding families in the United States with children diagnosed with ADHD spend an additional $15,000 per year (Sciberras et al., 2022). Individuals with ADHD are at higher risk of anxiety, depression, and overall report a lower quality of life (Kosse et al., 2017).

Pharmacological treatment options for ADHD include psychostimulant and non-psychostimulant medications, though clinical guidelines and efficacy and safety are still in debate (Cortese et al., 2018). A meta-analysis examined the available medications and found that while all but one were generally effective compared to placebo, they were also more efficacious in children than adults (Cortese et al., 2018). They also determined that amphetamines were more effective at all ages, but had varied effects across ages (Cortese et al., 2018). Overall, their results supported the use of methylphenidate for children and adolescents, and amphetamines for adults as the primary pharmacological choice (Cortese et al., 2018). These treatments can be highly effective initially for the majority of patients, but over time the side effects and low treatment acceptance may lead to the discontinuation of treatment (Biederman et al., 2019). Side effects can moreover include decreased appetite and sleep problems, as well as rarer cases of cardiac complications (Kosse et al., 2017). In children and adolescents, the non-adherence rate varies from 10 to 64% (Kosse et al., 2017).

ADHD is largely genetic, with a hereditability of 70–80%, and a smaller environmental risk factor (Demontis et al., 2019). There are also consistent structural and functional alterations related to ADHD symptoms in various brain regions, including the prefrontal cortex, basal ganglia, cerebellum, parietal cortex, and anterior cingulate cortex (Zaehle and Krauel, 2021). Neurochemically, individuals with ADHD have reduced levels of DA, NE, and GABA (Zaehle and Krauel, 2021). One study found that GABA concentrations were significantly lower in female and male children with ADHD compared to their typically developing counterparts (Edden et al., 2012). However, this study did not determine whether this reduction in GABA was due to the reduced density of GABAergic neurons or a reduced concentration of GABA with a normal neuronal density (Edden et al., 2012). Dopaminergic dysfunction in ADHD has been supported through animal studies and pharmacological treatments, like methylphenidate that blocks DA transporters at therapeutic doses, which could be linked to why ADHD patients are vulnerable to drug dependence (Véronneau-Veilleux et al., 2022).

The benefits of VNS for ADHD have not been experimentally explored, but its potential lies in its ability to increase NE and GABA concentrations in the brain. Studies have shown that VNS can have a protective effect on GABAergic neurons, as well as increased GABA transmission in the NTS in traumatic brain injury models (Zaehle and Krauel, 2021). In epilepsy patients, chronic VNS increases GABA receptor density in the frontal and temporal brain regions (Zaehle and Krauel, 2021). However, the main effects of VNS are believed to be mediated through the LC-NE system discussed in this review. It is known that NE can facilitate aspects of cognition that are lacking in individuals with ADHD, such as attention and behavioral flexibility (Zaehle and Krauel, 2021). Thus, modulation of NE by VNS may improve these behavioral and cognitive deficits. Furthermore, unlike currently approved pharmacological treatments, VNS is a well-accepted treatment modality with few side effects. The implementation of iVNS would remove the non-adherence component but add surgical risks and higher costs. Alternatively, non-invasive methods of VNS may be more practical and could provide a treatment that reduces the risk of drug dependence. Future studies should evaluate the efficacy of VNS in reducing ADHD symptoms to determine how patients may benefit.

## 8 Conclusions and future directions

While the inflammatory reflex encapsulates the anti-inflammatory effects of the VN in the periphery, there is a less well-understood mechanism for immunomodulation in the CNS. Microglia, the immune cells of the brain, are likely mediators of this anti-inflammatory action, notably regulated by neurochemical signaling. Surface receptors, such as α 7nAChR, β 2-adrenoreceptor, GABA_A_, and TrkB initiate the anti-inflammatory pathways to moderate microglial response. In line with this, we highlighted several studies demonstrating that VNS is a safe and effective treatment for epilepsy and depression. Although the specific mechanisms are unknown, the modulation of inflammation in the CNS and periphery, as well as modulation of various neurotransmitter pathways are possible routes for its actions. In both cases, microglia would play a critical role in controlling the release of inflammatory mediators. The potential for tVNS as a therapy for epilepsy and neurodevelopmental disorders like ASD, ADHD, and schizophrenia is promising. Through the suggested pathways, tVNS may be able to moderate neurotransmitter deficiencies and prevent the onset or progression of synaptic structural and functional alteration, microglial priming, and other microglial pathophysiological states. As a non-invasive approach, tVNS can increase accessibility to the therapy by reducing cost and risk. While it is still a developing therapeutic approach, it has been demonstrated to be tolerable, with effects comparable to iVNS. Future research is expected to focus on the role of microglia in the function of VNS and determine the efficacy of tVNS as an alternative therapy for neurodevelopmental disorders. As advocacy of VNS increases, it is becoming increasingly important to intensify efforts to understand the effect of VNS on microglia and their role in the immunomodulatory actions of VNS. In preclinical studies, microglia could be a critical marker to determine the neuroinflammatory response after VNS, able to elucidate both mechanism and effectiveness. In the meantime, it is expected that there will be changes in microglial ultrastructure, morphology, molecular signature, and sensome, which may contribute to modifying the inflammatory status of the CNS, as well as the function of nearby cells. Recent technical advancements, notably in 3-dimensional *in vivo* and *in situ* imaging for visualization of microglial phenotypes, as well as single-cell RNA sequencing and other omics techniques, are expected to enhance the understanding of the therapeutic effects of VNS throughout the CNS in the management of neurodevelopmental disorders.

Overall, this review highlights the potential of VNS as an alternative intervention for targeting microglia and for studies aiming to target microglia to treat neurodevelopmental disorders. Specifically, we reported that VNS has shown promise in normalizing the immune status, neuronal excitation, and brain plasticity. We discussed various findings and theoretical mechanisms of VNS’s parasympathomimetic therapeutic actions, which can be utilized in developing treatment strategies for different neurodevelopmental disorders.

## References

[ref1] Aalbers M. W. Klinkenberg S. Rijkers K. Verschuure P. Kessels A. Aldenkamp A.. (2012). The effects of Vagus nerve stimulation on pro-and anti-inflammatory cytokines in children with refractory epilepsy: An exploratory study. Neuroimmunomodulation 19, 352–358. doi: 10.1159/000341402, PMID: 23038102

[ref2] Addorisio M. E. Imperato G. H. de Vos A. F. Forti S. Goldstein R. S. Pavlov V. A.. (2019). Investigational treatment of rheumatoid arthritis with a vibrotactile device applied to the external ear. Bioelectron Med. 5:4. doi: 10.1186/s42234-019-0020-4 32232095 PMC7098240

[ref3] Agostoni E. Chinnock J. E. Daly M. D. B. Murray J. G. (1957). Functional and histological studies of the vagus nerve and its branches to the heart, lungs and abdominal viscera in the cat. J. Physiol. 135, 182–205. doi: 10.1113/jphysiol.1957.sp005703, PMID: 13398974 PMC1358921

[ref4] Ananth M. R. Rajebhosale P. Kim R. Talmage D. A. Role L. W. (2023). Basal forebrain cholinergic signalling: development, connectivity and roles in cognition. Nat. Rev. Neurosci. 24, 233–251. doi: 10.1038/s41583-023-00677-x 36823458 PMC10439770

[ref5] Andero R. Choi D. C. Ressler K. J. (2014). “Chapter six - BDNF–TrkB receptor regulation of distributed adult neural plasticity, memory formation, and psychiatric disorders” in Progress in molecular biology and translational science [internet]. eds. Khan Z. U. Muly E. C. (Cambridge, MA: Academic Press), 169–192. 10.1016/B978-0-12-420170-5.00006-4 24484701

[ref6] Andresen M. C. Yang M. Y. (1990). Non-NMDA receptors mediate sensory afferent synaptic transmission in medial nucleus tractus solitarius. Am. J. Phys. 259, H1307–H1311. doi: 10.1152/ajpheart.1990.259.4.H1307, PMID: 1977326

[ref7] Bachiller S. Jiménez-Ferrer I. Paulus A. Yang Y. Swanberg M. Deierborg T.. (2018). Microglia in neurological diseases: a road map to brain-disease dependent-inflammatory response. Front. Cell Neurosci. 12:12. doi: 10.3389/fncel.2018.00488, PMID: 30618635 PMC6305407

[ref8] Bachmann H. Vandemoortele B. Vermeirssen V. Carrette E. Vonck K. Boon P.. (2024). Vagus nerve stimulation enhances remyelination and decreases innate neuroinflammation in lysolecithin-induced demyelination. Brain Stimulat. 17, 575–587. doi: 10.1016/j.brs.2024.04.012, PMID: 38648972

[ref9] Badran B. W. Austelle C. W. (2022). The future is noninvasive: a brief review of the evolution and clinical utility of Vagus nerve stimulation. FOC 20, 3–7. doi: 10.1176/appi.focus.20210023, PMID: 35746934 PMC9063597

[ref10] Bansal V. Ryu S. Y. Lopez N. Allexan S. Krzyzaniak M. Eliceiri B.. (2012). Vagal stimulation modulates inflammation through a ghrelin mediated mechanism in traumatic brain injury. Inflammation 35, 214–220. doi: 10.1007/s10753-011-9307-7 21360048 PMC3282000

[ref11] Ben-Azu B. Adebayo O. G. Jarikre T. A. Oyovwi M. O. Edje K. E. Omogbiya I. A.. (2022). Taurine, an essential β-amino acid insulates against ketamine-induced experimental psychosis by enhancement of cholinergic neurotransmission, inhibition of oxidative/nitrergic imbalances, and suppression of COX-2/iNOS immunoreactions in mice. Metab. Brain Dis. 37, 2807–2826. doi: 10.1007/s11011-022-01075-5, PMID: 36057735

[ref12] Ben-Azu B. Aderibigbe A. O. Eneni A. E. O. Ajayi A. M. Umukoro S. Iwalewa E. O. (2018). Morin attenuates neurochemical changes and increased oxidative/Nitrergic stress in brains of mice exposed to ketamine: prevention and reversal of schizophrenia-like symptoms. Neurochem. Res. 43, 1745–1755. doi: 10.1007/s11064-018-2590-z, PMID: 29956036

[ref13] Ben-Azu B. Uruaka C. I. Ajayi A. M. Jarikre T. A. Nwangwa K. E. Chilaka K. C.. (2023). Reversal and preventive pleiotropic mechanisms involved in the antipsychotic-like effect of taurine, an essential β-amino acid in ketamine-induced experimental schizophrenia in mice. Neurochem. Res. 48, 816–829. doi: 10.1007/s11064-022-03808-5, PMID: 36350433

[ref14] Biederman J. Fried R. DiSalvo M. Storch B. Pulli A. Woodworth K. Y.. (2019). Evidence of low adherence to stimulant medication among children and youths with ADHD: An electronic health records study. Psychiatr. Serv. 70, 874–880. doi: 10.1176/appi.ps.201800515, PMID: 31242830

[ref15] Biggio F. Gorini G. Utzeri C. Olla P. Marrosu F. Mocchetti I.. (2009). Chronic vagus nerve stimulation induces neuronal plasticity in the rat hippocampus. Int. J. Neuropsychopharmacol. 12, 1209–1221. doi: 10.1017/S1461145709000200, PMID: 19309534 PMC2879889

[ref16] Bisht K. Sharma K. P. Lecours C. Gabriela Sánchez M. El Hajj H. Milior G.. (2016). Dark microglia: a new phenotype predominantly associated with pathological states. Glia 64, 826–839. doi: 10.1002/glia.22966, PMID: 26847266 PMC4949554

[ref17] Black B. Hunter S. Cottrell H. Dar R. Takahashi N. Ferguson B. J.. (2023). Remotely supervised at-home delivery of taVNS for autism spectrum disorder: feasibility and initial efficacy. Front. Psychiatry 14:14. Available from:. doi: 10.3389/fpsyt.2023.1238328 PMC10568329 37840787

[ref18] Bonaz B. Sinniger V. Pellissier S. (2017). The Vagus nerve in the neuro-immune Axis: implications in the pathology of the gastrointestinal tract. Front. Immunol. 8:8. doi: 10.3389/fimmu.2017.01452 29163522 PMC5673632

[ref19] Bonnycastle D. D. Giarman N. J. Paasonen M. K. (1957). Anticonvulsant compounds and 5-Hydroxy-tryptamine in rat brain. Br. J. Pharmacol. Chemother. 12, 228–231. doi: 10.1111/j.1476-5381.1957.tb00125.x 13446378 PMC1509665

[ref20] Borovikova L. V. Ivanova S. Zhang M. Yang H. Botchkina G. I. Watkins L. R.. (2000). Vagus nerve stimulation attenuates the systemic inflammatory response to endotoxin. Nature 405, 458–462. doi: 10.1038/35013070, PMID: 10839541

[ref21] Bravo J. A. Forsythe P. Chew M. V. Escaravage E. Savignac H. M. Dinan T. G.. (2011). Ingestion of Lactobacillus strain regulates emotional behavior and central GABA receptor expression in a mouse via the vagus nerve. Proc. Natl. Acad. Sci. USA 108, 16050–16055. doi: 10.1073/pnas.1102999108 21876150 PMC3179073

[ref22] Breit S. Kupferberg A. Rogler G. Hasler G. (2018). Vagus nerve as modulator of the brain–gut Axis in psychiatric and inflammatory disorders. Front. Psych. 9:44. doi: 10.3389/fpsyt.2018.00044, PMID: 29593576 PMC5859128

[ref23] Bromfield E. B. Cavazos J. E. Sirven J. I. (2006). “Basic mechanisms underlying seizures and epilepsy” in An introduction to epilepsy (Chicago, IL: American Epilepsy Society).

[ref24] Brougher J. Aziz U. Adari N. Chaturvedi M. Jules A. Shah I.. (2021). Self-Administration of Right Vagus Nerve Stimulation Activates Midbrain Dopaminergic Nuclei. Front. Neurosci. 15:15. Available from:. doi: 10.3389/fnins.2021.782786 PMC8716493 34975384

[ref25] Busse S. Busse M. Schiltz K. Bielau H. Gos T. Brisch R.. (2012). Different distribution patterns of lymphocytes and microglia in the hippocampus of patients with residual versus paranoid schizophrenia: further evidence for disease course-related immune alterations? Brain Behav. Immun. 26, 1273–1279. doi: 10.1016/j.bbi.2012.08.005, PMID: 22917959

[ref26] Capone F. Assenza G. Di Pino G. Musumeci G. Ranieri F. Florio L.. (2015). The effect of transcutaneous vagus nerve stimulation on cortical excitability. J. Neural Transm. 122, 679–685. doi: 10.1007/s00702-014-1299-7 25182412

[ref27] Chaouloff F. Hémar A. Manzoni O. (2007). Acute stress facilitates hippocampal CA1 metabotropic glutamate receptor-dependent Long-term depression. J. Neurosci. 27, 7130–7135. doi: 10.1523/JNEUROSCI.1150-07.2007 17611266 PMC6794579

[ref28] Charlton T. Prowse N. McFee A. Heiratifar N. Fortin T. Paquette C.. (2023). Brain-derived neurotrophic factor (BDNF) has direct anti-inflammatory effects on microglia. Front. Cell Neurosci. 17:17. doi: 10.3389/fncel.2023.1188672, PMID: 37404293 PMC10315457

[ref29] Chen H. Feng Z. Min L. Deng W. Tan M. Hong J.. (2022). Vagus nerve stimulation reduces Neuroinflammation through microglia polarization regulation to improve functional recovery after spinal cord injury. Front. Neurosci. 16:16. doi: 10.3389/fnins.2022.813472 PMC9022634 35464311

[ref30] Chen X. He X. Luo S. Feng Y. Liang F. Shi T.. (2018). Vagus nerve stimulation attenuates cerebral microinfarct and colitis-induced cerebral microinfarct aggravation in mice. Front. Neurol. 9:798. doi: 10.3389/fneur.2018.00798, PMID: 30319530 PMC6168656

[ref31] Colzato L. S. Sellaro R. Beste C. (2017). Darwin revisited: The vagus nerve is a causal element in controlling recognition of other’s emotions. Cortex 92, 95–102. doi: 10.1016/j.cortex.2017.03.017, PMID: 28460255

[ref32] Corsi-Zuelli F. M. Brognara F. da Silva Quirino G. F. Hiroki C. H. Fais R. S. Del-Ben C. M.. (2017). Neuroimmune interactions in schizophrenia: focus on Vagus nerve stimulation and activation of the Alpha-7 nicotinic acetylcholine receptor. Front. Immunol. 8:618. doi: 10.3389/fimmu.2017.00618 28620379 PMC5449450

[ref33] Cortese S. Adamo N. Giovane C. D. Mohr-Jensen C. Hayes A. J. Carucci S.. (2018). Comparative efficacy and tolerability of medications for attention-deficit hyperactivity disorder in children, adolescents, and adults: a systematic review and network meta-analysis. Lancet Psychiatry 5, 727–738. doi: 10.1016/S2215-0366(18)30269-4, PMID: 30097390 PMC6109107

[ref34] Cox M. A. Bassi C. Saunders M. E. Nechanitzky R. Morgado-Palacin I. Zheng C.. (2020). Beyond neurotransmission: acetylcholine in immunity and inflammation. J. Intern. Med. 287, 120–133. doi: 10.1111/joim.13006, PMID: 31710126

[ref35] Danielsson S. Viggedal G. Gillberg C. Olsson I. (2008). Lack of effects of vagus nerve stimulation on drug-resistant epilepsy in eight pediatric patients with autism spectrum disorders: a prospective 2-year follow-up study. Epilepsy Behav. 12, 298–304. doi: 10.1016/j.yebeh.2007.10.007, PMID: 18053767

[ref36] Davis S. F. Derbenev A. V. Williams K. W. Glatzer N. R. Smith B. N. (2004). Excitatory and inhibitory local circuit input to the rat dorsal motor nucleus of the vagus originating from the nucleus tractus solitarius. Brain Res. 1017, 208–217. doi: 10.1016/j.brainres.2004.05.049, PMID: 15261116 PMC3761086

[ref37] Dawson J. Liu C. Y. Francisco G. E. Cramer S. C. Wolf S. L. Dixit A.. (2021). Vagus nerve stimulation paired with rehabilitation for upper limb motor function after ischaemic stroke (VNS-REHAB): a randomised, blinded, pivotal, device trial. Lancet 397, 1545–1553. doi: 10.1016/S0140-6736(21)00475-X, PMID: 33894832 PMC8862193

[ref38] De Herdt V. Bogaert S. Bracke K. R. Raedt R. De Vos M. Vonck K.. (2009). Effects of vagus nerve stimulation on pro-and anti-inflammatory cytokine induction in patients with refractory epilepsy. J. Neuroimmunol. 214, 104–108. doi: 10.1016/j.jneuroim.2009.06.008, PMID: 19608283

[ref39] de Jonge W. J. van der Zanden E. P. The F. O. Bijlsma M. F. van Westerloo D. J. Bennink R. J.. (2005). Stimulation of the vagus nerve attenuates macrophage activation by activating the Jak2-STAT3 signaling pathway. Nat. Immunol. 6, 844–851. doi: 10.1038/ni1229, PMID: 16025117

[ref40] De Picker L. J. Victoriano G. M. Richards R. Gorvett A. J. Lyons S. Buckland G. R.. (2021). Immune environment of the brain in schizophrenia and during the psychotic episode: a human post-mortem study. Brain Behav. Immun. 97, 319–327. doi: 10.1016/j.bbi.2021.07.017, PMID: 34339805 PMC8475749

[ref41] Demontis D. Walters R. K. Martin J. Mattheisen M. Als T. D. Agerbo E.. (2019). Discovery of the first genome-wide significant risk loci for attention deficit/hyperactivity disorder. Nat. Genet. 51, 63–75. doi: 10.1038/s41588-018-0269-7 30478444 PMC6481311

[ref42] Devoto P. Flore G. Saba P. Fà M. Gessa G. L. (2005). Co-release of noradrenaline and dopamine in the cerebral cortex elicited by single train and repeated train stimulation of the locus coeruleus. BMC Neurosci. 6:31. doi: 10.1186/1471-2202-6-31, PMID: 15865626 PMC1134661

[ref43] Dolezalova I. Koritakova E. Souckova L. Chrastina J. Chladek J. Stepanova R.. (2022). Prediction of vagal nerve stimulation efficacy in drug-resistant epilepsy (PRECISE): prospective study for pre-implantation prediction/study design. Front. Neurol. 13:13. doi: 10.3389/fneur.2022.839163 PMC8979018 35386419

[ref44] Dorr A. E. Debonnel G. (2006). Effect of vagus nerve stimulation on serotonergic and noradrenergic transmission. J. Pharmacol. Exp. Ther. 318, 890–898. doi: 10.1124/jpet.106.104166, PMID: 16690723

[ref45] DSM Library. (2024) Diagnostic and statistical manual of mental disorders. Available online at: https://dsm.psychiatryonline.org/doi/book/10.1176/appi.books.9780890425596

[ref46] Edden R. A. E. Crocetti D. Zhu H. Gilbert D. L. Mostofsky S. H. (2012). Reduced GABA concentration in attention-deficit/hyperactivity disorder. Arch. Gen. Psychiatry 69, 750–753. doi: 10.1001/archgenpsychiatry.2011.2280 22752239 PMC3970207

[ref47] Eickhoff S. Franzen L. Korda A. Rogg H. Trulley V. N. Borgwardt S.. (2022). The basal forebrain cholinergic nuclei and their relevance to schizophrenia and other psychotic disorders. Front. Psych. 13:909961. doi: 10.3389/fpsyt.2022.909961, PMID: 35873225 PMC9299093

[ref48] Ek M. Kurosawa M. Lundeberg T. Ericsson A. (1998). Activation of vagal afferents after intravenous injection of interleukin-1beta: role of endogenous prostaglandins. J. Neurosci. 18, 9471–9479. doi: 10.1523/JNEUROSCI.18-22-09471.1998, PMID: 9801384 PMC6792875

[ref49] Elenkov I. J. Wilder R. L. Chrousos G. P. Vizi E. S. (2000). The sympathetic nerve--an integrative interface between two supersystems: the brain and the immune system. Pharmacol. Rev. 52, 595–638, PMID: 11121511

[ref50] Engineer C. T. Hays S. A. Kilgard M. P. (2017). Vagus nerve stimulation as a potential adjuvant to behavioral therapy for autism and other neurodevelopmental disorders. J. Neurodev. Disord. 9:20. doi: 10.1186/s11689-017-9203-z, PMID: 28690686 PMC5496407

[ref51] Espana A. Seth H. Jézéquel J. Huang T. Bouchet D. Lepleux M.. (2021). Alteration of NMDA receptor trafficking as a cellular hallmark of psychosis. Transl. Psychiatry 11, 444–411. doi: 10.1038/s41398-021-01549-7, PMID: 34462417 PMC8405679

[ref52] Fetzer S. Dibué M. Nagel A. M. Trollmann R. (2021). A systematic review of magnetic resonance imaging in patients with an implanted vagus nerve stimulation system. Neuroradiology 63, 1407–1417. doi: 10.1007/s00234-021-02705-y, PMID: 33846830 PMC8376717

[ref53] Fisher B. DesMarteau J. A. Koontz E. H. Wilks S. J. Melamed S. E. (2020). Responsive Vagus nerve stimulation for drug resistant epilepsy: a review of new features and practical guidance for advanced practice providers. Front. Neurol. 11:610379. doi: 10.3389/fneur.2020.610379 33584511 PMC7874068

[ref54] Foley J. O. DuBois F. S. (1937). Quantitative studies of the vagus nerve in the cat. I. The ratio of sensory to motor fibers. J. Comp. Neurol. 67, 49–67. doi: 10.1002/cne.900670104

[ref55] Follesa P. Biggio F. Gorini G. Caria S. Talani G. Dazzi L.. (2007). Vagus nerve stimulation increases norepinephrine concentration and the gene expression of BDNF and bFGF in the rat brain. Brain Res. 1179, 28–34. doi: 10.1016/j.brainres.2007.08.045, PMID: 17920573

[ref56] Freedman R. Hall M. Adler L. E. Leonard S. (1995). Evidence in postmortem brain tissue for decreased numbers of hippocampal nicotinic receptors in schizophrenia. Biol. Psychiatry 38, 22–33. doi: 10.1016/0006-3223(94)00252-X 7548469

[ref57] Freedman R. Olincy A. Buchanan R. W. Harris J. G. Gold J. M. Johnson L.. (2008). Initial phase 2 trial of a nicotinic agonist in schizophrenia. Am. J. Psychiatry 165, 1040–1047. doi: 10.1176/appi.ajp.2008.07071135, PMID: 18381905 PMC3746983

[ref58] Friedman B. A. Srinivasan K. Ayalon G. Meilandt W. J. Lin H. Huntley M. A.. (2018). Diverse brain myeloid expression profiles reveal distinct microglial activation states and aspects of Alzheimer’s disease not evident in mouse models. Cell Rep. 22, 832–847. doi: 10.1016/j.celrep.2017.12.066, PMID: 29346778

[ref59] Frisén J. Verge V. M. Fried K. Risling M. Persson H. Trotter J.. (1993). Characterization of glial trkB receptors: differential response to injury in the central and peripheral nervous systems. Proc. Natl. Acad. Sci. USA 90, 4971–4975. doi: 10.1073/pnas.90.11.4971, PMID: 8389459 PMC46635

[ref60] Fukuda M. Matsuo T. Fujimoto S. Kashii H. Hoshino A. Ishiyama A.. (2024). Vagus nerve stimulation therapy for drug-resistant epilepsy in children—a literature review. J. Clin. Med. 13:780. doi: 10.3390/jcm13030780, PMID: 38337474 PMC10856244

[ref61] Furmaga H. Carreno F. R. Frazer A. (2012). Vagal nerve stimulation rapidly activates brain-derived neurotrophic factor receptor TrkB in rat brain. PLoS One 7:e34844. doi: 10.1371/journal.pone.0034844, PMID: 22563458 PMC3341395

[ref62] Furman D. Campisi J. Verdin E. Carrera-Bastos P. Targ S. Franceschi C.. (2019). Chronic inflammation in the etiology of disease across the life span. Nat. Med. 25, 1822–1832. doi: 10.1038/s41591-019-0675-0 31806905 PMC7147972

[ref63] Genovese M. C. Gaylis N. B. Sikes D. Kivitz A. Lewis Horowitz D. Peterfy C.. (2020). Safety and efficacy of neurostimulation with a miniaturised vagus nerve stimulation device in patients with multidrug-refractory rheumatoid arthritis: a two-stage multicentre, randomised pilot study. Lancet Rheumatol. 2, e527–e538. doi: 10.1016/S2665-9913(20)30172-7, PMID: 38273617

[ref64] Ginhoux F. Greter M. Leboeuf M. Nandi S. See P. Gokhan S.. (2010). Fate mapping analysis reveals that adult microglia derive from primitive macrophages. Science 330, 841–845. doi: 10.1126/science.1194637, PMID: 20966214 PMC3719181

[ref65] Giorgi F. S. Pizzanelli C. Biagioni F. Murri L. Fornai F. (2004). The role of norepinephrine in epilepsy: from the bench to the bedside. Neurosci. Biobehav. Rev. 28, 507–524. doi: 10.1016/j.neubiorev.2004.06.008, PMID: 15465138

[ref66] Gober R. Ardalan M. Shiadeh S. M. J. Duque L. Garamszegi S. P. Ascona M.. (2022). Microglia activation in postmortem brains with schizophrenia demonstrates distinct morphological changes between brain regions. Brain Pathol. 32:e13003. doi: 10.1111/bpa.13003, PMID: 34297453 PMC8713533

[ref67] Goehler L. E. Gaykema R. P. Hansen M. K. Anderson K. Maier S. F. Watkins L. R. (2000). Vagal immune-to-brain communication: a visceral chemosensory pathway. Auton. Neurosci. 85, 49–59. doi: 10.1016/S1566-0702(00)00219-8, PMID: 11189026

[ref68] Goehler L. E. Gaykema R. P. A. Nguyen K. T. Lee J. E. Tilders F. J. H. Maier S. F.. (1999). Interleukin-1β in immune cells of the abdominal Vagus nerve: a link between the immune and nervous systems? J. Neurosci. 19, 2799–2806. doi: 10.1523/JNEUROSCI.19-07-02799.1999, PMID: 10087091 PMC6786076

[ref69] Goggins E. Mitani S. Tanaka S. (2022). Clinical perspectives on vagus nerve stimulation: present and future. Clin. Sci. (Lond.) 136, 695–709. doi: 10.1042/CS20210507, PMID: 35536161 PMC9093220

[ref70] Gombkoto P. Gielow M. Varsanyi P. Chavez C. Zaborszky L. (2021). Contribution of the basal forebrain to corticocortical network interactions. Brain Struct. Funct. 226, 1803–1821. doi: 10.1007/s00429-021-02290-z, PMID: 34021788 PMC8203523

[ref71] Grando S. A. Kawashima K. Wessler I. (2003). Introduction: the non-neuronal cholinergic system in humans. Life Sci. 72, 2009–2012. doi: 10.1016/S0024-3205(03)00063-8, PMID: 12628450

[ref72] Guerrini R. (2006). Epilepsy in children. Lancet 367, 499–524. doi: 10.1016/S0140-6736(06)68182-8 16473127

[ref73] Ha S. Sohn I. J. Kim N. Sim H. J. Cheon K. A. (2015). Characteristics of brains in autism Spectrum disorder: structure, function and connectivity across the lifespan. Exp Neurobiol. 24, 273–284. doi: 10.5607/en.2015.24.4.273, PMID: 26713076 PMC4688328

[ref74] Haddad P. M. Correll C. U. (2018). The acute efficacy of antipsychotics in schizophrenia: a review of recent meta-analyses. Ther Adv Psychopharmacol. 8, 303–318. doi: 10.1177/2045125318781475, PMID: 30344997 PMC6180374

[ref75] Hajtovic S. LoPresti M. A. Zhang L. Katlowitz K. A. Kizek D. J. Lam S. (2022). The role of vagus nerve stimulation in genetic etiologies of drug-resistant epilepsy: a meta-analysis. J. Neurosurg. Pediatr. 29, 667–680. doi: 10.3171/2022.1.PEDS222 35303699

[ref76] Han Z. Shen F. He Y. Degos V. Camus M. Maze M.. (2014). Activation of α-7 nicotinic acetylcholine receptor reduces ischemic stroke injury through reduction of pro-inflammatory macrophages and oxidative stress. PLoS One 9:e105711. doi: 10.1371/journal.pone.0105711, PMID: 25157794 PMC4144901

[ref77] Han W. Tellez L. A. Perkins M. H. Perez I. O. Qu T. Ferreira J.. (2018). A neural circuit for gut-induced reward. Cell 175, 665–678.e23. doi: 10.1016/j.cell.2018.08.049 30245012 PMC6195474

[ref78] Hansen M. K. O’Connor K. A. Goehler L. E. Watkins L. R. Maier S. F. (2001). The contribution of the vagus nerve in interleukin-1β-induced fever is dependent on dose. Am. J. Phys. Regul. Integr. Comp. Phys. 280, R929–R934. doi: 10.1152/ajpregu.2001.280.4.R929 11247812

[ref79] Hao J. Simard A. R. Turner G. H. Wu J. Whiteaker P. Lukas R. J.. (2011). Attenuation of CNS inflammatory responses by nicotine involves α7 and non-α7 nicotinic receptors. Exp. Neurol. 227, 110–119. doi: 10.1016/j.expneurol.2010.09.020, PMID: 20932827 PMC3019302

[ref80] Hara Y. Ago Y. Higuchi M. Hasebe S. Nakazawa T. Hashimoto H.. (2017). Oxytocin attenuates deficits in social interaction but not recognition memory in a prenatal valproic acid-induced mouse model of autism. Horm. Behav. 96, 130–136. doi: 10.1016/j.yhbeh.2017.09.013, PMID: 28942000

[ref81] Hasan A. Wolff-Menzler C. Pfeiffer S. Falkai P. Weidinger E. Jobst A.. (2015). Transcutaneous noninvasive vagus nerve stimulation (tVNS) in the treatment of schizophrenia: a bicentric randomized controlled pilot study. Eur. Arch. Psychiatry Clin. Neurosci. 265, 589–600. doi: 10.1007/s00406-015-0618-9, PMID: 26210303

[ref82] Hassert D. L. Miyashita T. Williams C. L. (2004). The effects of peripheral vagal nerve stimulation at a memory-modulating intensity on norepinephrine output in the basolateral amygdala. Behav. Neurosci. 118, 79–88. doi: 10.1037/0735-7044.118.1.79, PMID: 14979784

[ref83] Hayes L. N. An K. Carloni E. Li F. Vincent E. Trippaers C.. (2022). Prenatal immune stress blunts microglia reactivity, impairing neurocircuitry. Nature 610, 327–334. doi: 10.1038/s41586-022-05274-z, PMID: 36171283 PMC13436803

[ref84] Hays S. A. Rennaker R. L. Kilgard M. P. (2013). Targeting plasticity with Vagus nerve stimulation to treat neurological disease. Prog. Brain Res. 207, 275–299. doi: 10.1016/B978-0-444-63327-9.00010-2, PMID: 24309259 PMC4615598

[ref85] Hickman S. E. Kingery N. D. Ohsumi T. K. Borowsky M. L. Wang L. C. Means T. K.. (2013). The microglial sensome revealed by direct RNA sequencing. Nat. Neurosci. 16, 1896–1905. doi: 10.1038/nn.3554, PMID: 24162652 PMC3840123

[ref86] Honey D. Wosnitzka E. Klann E. Weinhard L. (2022). Analysis of microglial BDNF function and expression in the motor cortex. Front. Cell Neurosci. 16:16. doi: 10.3389/fncel.2022.961276, PMID: 36726454 PMC9885322

[ref87] Hong G. S. Zillekens A. Schneiker B. Pantelis D. de Jonge W. J. Schaefer N.. (2019). Non-invasive transcutaneous auricular vagus nerve stimulation prevents postoperative ileus and endotoxemia in mice. Neurogastroenterol. Motility 31:e13501. doi: 10.1111/nmo.13501 30406957

[ref88] Howland R. H. (2014). Vagus nerve stimulation. Curr. Behav. Neurosci. Rep. 1, 64–73. doi: 10.1007/s40473-014-0010-5 24834378 PMC4017164

[ref89] Hulsey D. R. Hays S. A. Khodaparast N. Ruiz A. Das P. Rennaker R. L.. (2016). Reorganization of motor cortex by Vagus nerve stimulation requires cholinergic innervation. Brain Stimul. 9, 174–181. doi: 10.1016/j.brs.2015.12.007, PMID: 26822960 PMC4789078

[ref90] Hyvärinen P. Yrttiaho S. Lehtimäki J. Ilmoniemi R. J. Mäkitie A. Ylikoski J.. (2015). Transcutaneous vagus nerve stimulation modulates tinnitus-related beta-and gamma-band activity. Ear Hear. 36, e76–e85. doi: 10.1097/AUD.0000000000000123, PMID: 25437140

[ref91] Ikawa D. Makinodan M. Iwata K. Ohgidani M. Kato T. A. Yamashita Y.. (2017). Microglia-derived neuregulin expression in psychiatric disorders. Brain Behav. Immun. 61, 375–385. doi: 10.1016/j.bbi.2017.01.003, PMID: 28089559

[ref92] Ji T. Yang Z. Liu Q. Liao J. Yin F. Chen Y.. (2019). Vagus nerve stimulation for pediatric patients with intractable epilepsy between 3 and 6 years of age: study protocol for a double-blind, randomized control trial. Trials 20:44. doi: 10.1186/s13063-018-3087-4, PMID: 30642370 PMC6332620

[ref93] Jiang N. M. Cowan M. Moonah S. N. Petri W. A. (2018). The impact of systemic inflammation on neurodevelopment. Trends Mol. Med. 24, 794–804. doi: 10.1016/j.molmed.2018.06.008 30006148 PMC6110951

[ref94] Jiang Y. Li L. Liu B. Zhang Y. Chen Q. Li C. (2014). Vagus nerve stimulation attenuates cerebral ischemia and reperfusion injury via endogenous cholinergic pathway in rat. PLoS One 9:e102342. doi: 10.1371/journal.pone.0102342, PMID: 25036185 PMC4103831

[ref95] Jiang Y. Li L. Liu B. Zhang Y. Chen Q. Li C. (2015). PPARγ upregulation induced by Vagus nerve stimulation exerts anti-inflammatory effect in cerebral ischemia/reperfusion rats. Med. Sci. Monit. 21, 268–275. doi: 10.12659/MSM.891407, PMID: 25619160 PMC4310716

[ref96] Jin Z. Dong J. Wang Y. Liu Y. (2023). Exploring the potential of vagus nerve stimulation in treating brain diseases: a review of immunologic benefits and neuroprotective efficacy. Eur. J. Med. Res. 28:444. doi: 10.1186/s40001-023-01439-2, PMID: 37853458 PMC10585738

[ref97] Jin Y. Kong J. (2017). Transcutaneous Vagus nerve stimulation: a promising method for treatment of autism Spectrum disorders. Front. Neurosci. 10:609. doi: 10.3389/fnins.2016.00609 28163670 PMC5247460

[ref98] Juárez Olguín H. Calderón Guzmán D. Hernández García E. Barragán M. G. (2016). The Role of dopamine and its dysfunction as a consequence of oxidative stress. Oxidative Med. Cell. Longev. 2016:9730467. doi: 10.1155/2016/9730467, PMID: 26770661 PMC4684895

[ref99] Kaczmarczyk R. Tejera D. Simon B. J. Heneka M. T. (2018). Microglia modulation through external vagus nerve stimulation in a murine model of Alzheimer’s disease. J. Neurochem. 146, 76–85. doi: 10.1111/jnc.14284 29266221

[ref100] Kapadia R. Yi J. H. Vemuganti R. (2008). Mechanisms of anti-inflammatory and neuroprotective actions of PPAR-gamma agonists. Front. Biosci. 13, 1813–1826. doi: 10.2741/2802 17981670 PMC2734868

[ref101] Karege F. Bondolfi G. Gervasoni N. Schwald M. Aubry J. M. Bertschy G. (2005). Low brain-derived neurotrophic factor (BDNF) levels in serum of depressed patients probably results from lowered platelet BDNF release unrelated to platelet reactivity. Biol. Psychiatry 57, 1068–1072. doi: 10.1016/j.biopsych.2005.01.008, PMID: 15860348

[ref102] Kaya M. Gurses C. Kalayci R. Ekizoglu O. Ahishali B. Orhan N.. (2008). Morphological and functional changes of blood-brain barrier in kindled rats with cortical dysplasia. Brain Res. 1208, 181–191. doi: 10.1016/j.brainres.2008.02.101 18395195

[ref103] Kaya M. Orhan N. Karabacak E. Bahceci M. B. Arican N. Ahishali B.. (2013). Vagus nerve stimulation inhibits seizure activity and protects blood-brain barrier integrity in kindled rats with cortical dysplasia. Life Sci. 92, 289–297. doi: 10.1016/j.lfs.2013.01.009, PMID: 23333826

[ref104] Kelly M. J. Breathnach C. Tracey K. J. Donnelly S. C. (2022). Manipulation of the inflammatory reflex as a therapeutic strategy. Cell Reports Med. 3:100696. doi: 10.1016/j.xcrm.2022.100696, PMID: 35858588 PMC9381415

[ref105] Keute M. Wienke C. Ruhnau P. Zaehle T. (2021). Effects of transcutaneous vagus nerve stimulation (tVNS) on beta and gamma brain oscillations. Cortex 140, 222–231. doi: 10.1016/j.cortex.2021.04.004, PMID: 34015727

[ref106] Kim T. H. Kim S. J. Lee S. M. (2014). Stimulation of the α7 nicotinic acetylcholine receptor protects against sepsis by inhibiting toll-like receptor via phosphoinositide 3-kinase activation. J. Infect. Dis. 209, 1668–1677. doi: 10.1093/infdis/jit669, PMID: 24298024

[ref107] Klarer M. Arnold M. Günther L. Winter C. Langhans W. Meyer U. (2014). Gut vagal afferents differentially modulate innate anxiety and learned fear. J. Neurosci. 34, 7067–7076. doi: 10.1523/JNEUROSCI.0252-14.2014 24849343 PMC6608191

[ref108] Klarer M. Krieger J. P. Richetto J. Weber-Stadlbauer U. Günther L. Winter C.. (2018). Abdominal vagal afferents modulate the brain transcriptome and behaviors relevant to schizophrenia. J. Neurosci. 38, 1634–1647. doi: 10.1523/JNEUROSCI.0813-17.2017, PMID: 29326171 PMC6705869

[ref109] Komori T. Okamura K. Ikehara M. Yamamuro K. Endo N. Okumura K.. (2024). Brain-derived neurotrophic factor from microglia regulates neuronal development in the medial prefrontal cortex and its associated social behavior. Mol. Psychiatry 29, 1338–1349. doi: 10.1038/s41380-024-02413-y 38243072 PMC11189755

[ref110] Koopman F. A. Chavan S. S. Miljko S. Grazio S. Sokolovic S. Schuurman P. R.. (2016). Vagus nerve stimulation inhibits cytokine production and attenuates disease severity in rheumatoid arthritis. Proc. Natl. Acad. Sci. 113, 8284–8289. doi: 10.1073/pnas.1605635113, PMID: 27382171 PMC4961187

[ref111] Koskuvi M. Pörsti E. Hewitt T. Räsänen N. Wu Y. C. Trontti K.. (2024). Genetic contribution to microglial activation in schizophrenia. Mol. Psychiatry 22, 1–12. doi: 10.1038/s41380-024-02529-1 PMC11420079 38519640

[ref112] Kosse R. C. Bouvy M. L. Philbert D. de Vries T. W. Koster E. S. (2017). Attention-deficit/hyperactivity disorder medication use in adolescents: The Patient’s perspective. J. Adolesc. Health 61, 619–625. doi: 10.1016/j.jadohealth.2017.05.027 28899641

[ref113] Koukouli F. Rooy M. Tziotis D. Sailor K. A. O’Neill H. C. Levenga J.. (2017). Nicotine reverses hypofrontality in animal models of addiction and schizophrenia. Nat. Med. 23, 347–354. doi: 10.1038/nm.4274, PMID: 28112735 PMC5819879

[ref114] Krahl S. E. Clark K. B. Smith D. C. Browning R. A. (1998). Locus coeruleus lesions suppress the seizure-attenuating effects of vagus nerve stimulation. Epilepsia 39, 709–714. doi: 10.1111/j.1528-1157.1998.tb01155.x, PMID: 9670898

[ref115] Lampros M. Vlachos N. Zigouris A. Voulgaris S. Alexiou G. A. (2021). Transcutaneous Vagus nerve stimulation (t-VNS) and epilepsy: a systematic review of the literature. Seizure 91, 40–48. doi: 10.1016/j.seizure.2021.05.017, PMID: 34090145

[ref116] Landau A. M. Dyve S. Jakobsen S. Alstrup A. K. O. Gjedde A. Doudet D. J. (2015). Acute vagal nerve stimulation lowers α2 adrenoceptor availability: possible mechanism of therapeutic action. Brain Stimul. 8, 702–707. doi: 10.1016/j.brs.2015.02.003, PMID: 25758422

[ref117] Laureys G. Gerlo S. Spooren A. Demol F. De Keyser J. Aerts J. L. (2014). β2-adrenergic agonists modulate TNF-α induced astrocytic inflammatory gene expression and brain inflammatory cell populations. J. Neuroinflammation 11:21. doi: 10.1186/1742-2094-11-21, PMID: 24479486 PMC3942172

[ref118] Laursen T. M. Munk-Olsen T. Vestergaard M. (2012). Life expectancy and cardiovascular mortality in persons with schizophrenia. Curr. Opin. Psychiatry 25, 83–88. doi: 10.1097/YCO.0b013e32835035ca 22249081

[ref119] Leonard S. Breese C. Adams C. Benhammou K. Gault J. Stevens K.. (2000). Smoking and schizophrenia: abnormal nicotinic receptor expression. Eur. J. Pharmacol. 393, 237–242. doi: 10.1016/S0014-2999(00)00035-2, PMID: 10771019

[ref120] Leuzinger W. Baker A. L. Cauvin E. (1968). Acetylcholinesterase. II. Crystallization, absorption spectra, isoionic point. Proc. Natl. Acad. Sci. USA 59, 620–623. doi: 10.1073/pnas.59.2.620, PMID: 5238989 PMC224717

[ref121] Li L. Liu Z. Jiang Y. Y. Shen W. X. Peng Y. P. Qiu Y. H. (2019). Acetylcholine suppresses microglial inflammatory response via α7nAChR to protect hippocampal neurons. JIN. 18, 51–56. doi: 10.31083/j.jin.2019.01.114 31091848

[ref122] Li Y. Owyang C. (2003). Musings on the wanderer: What’s new in our understanding of Vago-vagal reflexes? V. Remodeling of vagus and enteric neural circuitry after vagal injury. American journal of physiology-gastrointestinal and liver. Physiology 285, G461–G469. doi: 10.1152/ajpgi.00119.2003 12909562

[ref123] Li X. Wei N. Song J. Liu J. Yuan J. Song R.. (2023). The global burden of schizophrenia and the impact of urbanization during 1990-2019: An analysis of the global burden of disease study 2019. Environ. Res. 232:116305. doi: 10.1016/j.envres.2023.116305, PMID: 37268204

[ref124] Liu T. T. Chen S. P. Wang S. J. Yen J. C. (2024). Vagus nerve stimulation inhibits cortical spreading depression via glutamate-dependent TrkB activation mechanism in the nucleus tractus solitarius. Cephalalgia 44:03331024241230466. doi: 10.1177/03331024241230466, PMID: 38329067

[ref125] Liu H. Leak R. K. Hu X. (2016). Neurotransmitter receptors on microglia. Stroke Vasc Neurol. 1, 52–58. doi: 10.1136/svn-2016-000012 28959464 PMC5435193

[ref126] Lockard J. S. Congdon W. C. DuCharme L. L. (1990). Feasibility and safety of vagal stimulation in monkey model. Epilepsia 31, S20–S26. doi: 10.1111/j.1528-1157.1990.tb05844.x 2226362

[ref127] Majoie H. J. M. Rijkers K. Berfelo M. W. Hulsman J. Myint A. Schwarz M.. (2011). Vagus nerve stimulation in refractory epilepsy: effects on pro-and anti-inflammatory cytokines in peripheral blood. Neuroimmunomodulation 18, 52–56. doi: 10.1159/000315530, PMID: 20639683

[ref128] Manta S. Dong J. Debonnel G. Blier P. (2009a). Enhancement of the function of rat serotonin and norepinephrine neurons by sustained vagus nerve stimulation. J. Psychiatry Neurosci. 34, 272–280, PMID: 19568478 PMC2702444

[ref129] Manta S. Dong J. Debonnel G. Blier P. (2009b). Optimization of vagus nerve stimulation parameters using the firing activity of serotonin neurons in the rat dorsal raphe. Eur. Neuropsychopharmacol. 19, 250–255. doi: 10.1016/j.euroneuro.2008.12.001, PMID: 19150228

[ref130] Marotta R. Risoleo M. C. Messina G. Parisi L. Carotenuto M. Vetri L.. (2020). The neurochemistry of autism. Brain Sci. 10:163. doi: 10.3390/brainsci10030163 32182969 PMC7139720

[ref131] Marrosu F. Serra A. Maleci A. Puligheddu M. Biggio G. Piga M. (2003). Correlation between GABA(a) receptor density and vagus nerve stimulation in individuals with drug-resistant partial epilepsy. Epilepsy Res. 55, 59–70. doi: 10.1016/S0920-1211(03)00107-4, PMID: 12948617

[ref132] McEwen B. S. (2017). Neurobiological and systemic effects of chronic stress. Chronic Stress 1:2470547017692328. doi: 10.1177/2470547017692328, PMID: 28856337 PMC5573220

[ref133] McGehee D. S. Heath M. J. Gelber S. Devay P. Role L. W. (1995). Nicotine enhancement of fast excitatory synaptic transmission in CNS by presynaptic receptors. Science 269, 1692–1696. doi: 10.1126/science.7569895, PMID: 7569895

[ref134] Meneses G. Bautista M. Florentino A. Díaz G. Acero G. Besedovsky H.. (2016). Electric stimulation of the vagus nerve reduced mouse neuroinflammation induced by lipopolysaccharide. J. Inflamm. 13:33. doi: 10.1186/s12950-016-0140-5 PMC5086408 27807399

[ref135] Milby A. H. Halpern C. H. Baltuch G. H. (2009). Vagus nerve stimulation in the treatment of refractory epilepsy. Neurotherapeutics 6, 228–237. doi: 10.1016/j.nurt.2009.01.010, PMID: 19332314 PMC5084198

[ref136] Min D. K. Tuor U. I. Chelikani P. K. (2011). Gastric distention induced functional magnetic resonance signal changes in the rodent brain. Neuroscience 179, 151–158. doi: 10.1016/j.neuroscience.2011.01.051, PMID: 21284950

[ref137] Miyamoto A. Wake H. Ishikawa A. W. Eto K. Shibata K. Murakoshi H.. (2016). Microglia contact induces synapse formation in developing somatosensory cortex. Nat. Commun. 7:12540. doi: 10.1038/ncomms12540, PMID: 27558646 PMC5007295

[ref138] Miyatsu T. Oviedo V. Reynaga J. Karuzis V. P. Martinez D. O’Rourke P.. (2024). Transcutaneous cervical vagus nerve stimulation enhances second-language vocabulary acquisition while simultaneously mitigating fatigue and promoting focus. Sci. Rep. 14:17177. doi: 10.1038/s41598-024-68015-4, PMID: 39060415 PMC11282064

[ref139] Monier A. Adle-Biassette H. Delezoide A. L. Evrard P. Gressens P. Verney C. (2007). Entry and distribution of microglial cells in human embryonic and fetal cerebral cortex. J. Neuropathol. Exp. Neurol. 66, 372–382. doi: 10.1097/nen.0b013e3180517b46, PMID: 17483694

[ref140] Moret C. Briley M. (2011). The importance of norepinephrine in depression. Neuropsychiatr. Dis. Treat. 7, 9–13. doi: 10.2147/NDT.S19619, PMID: 21750623 PMC3131098

[ref141] Moshé S. L. Perucca E. Ryvlin P. Tomson T. (2015). Epilepsy: new advances. Lancet 385, 884–898. doi: 10.1016/S0140-6736(14)60456-6 25260236

[ref142] Müller N. Bechter K. (2013). The mild encephalitis concept for psychiatric disorders revisited in the light of current psychoneuroimmunological findings. Neurol. Psychiatry Brain Res. 19, 87–101. doi: 10.1016/j.npbr.2013.04.004

[ref143] Murphy M. D. Heller E. A. (2022). Convergent actions of stress and stimulants via epigenetic regulation of neural circuitry. Trends Neurosci. 45, 955–967. doi: 10.1016/j.tins.2022.10.001, PMID: 36280459 PMC9671852

[ref144] Murray K. Rude K. M. Sladek J. Reardon C. (2021). Divergence of neuroimmune circuits activated by afferent and efferent vagal nerve stimulation in the regulation of inflammation. J. Physiol. 599, 2075–2084. doi: 10.1113/JP281189, PMID: 33491187 PMC8016722

[ref145] Namgung U. Kim K. J. Jo B. G. Park J. M. (2022). Vagus nerve stimulation modulates hippocampal inflammation caused by continuous stress in rats. J. Neuroinflammation 19, 1–15. doi: 10.1186/s12974-022-02396-z 35109857 PMC8812005

[ref146] National Institute of Neurological Disorders and Stroke. (2024). Epilepsy and Seizures. Available online at: https://www.ninds.nih.gov/health-information/disorders/epilepsy-and-seizures

[ref147] Nesbitt A. D. Marin J. C. A. Tompkins E. Ruttledge M. H. Goadsby P. J. (2015). Initial use of a novel noninvasive vagus nerve stimulator for cluster headache treatment. Neurology 84, 1249–1253. doi: 10.1212/WNL.0000000000001394, PMID: 25713002

[ref148] Nguyen K. D. Amerio A. Aguglia A. Magnani L. Parise A. Conio B.. (2023). Microglia and other cellular mediators of immunological dysfunction in schizophrenia: a narrative synthesis of clinical findings. Cells 12:2099. doi: 10.3390/cells12162099, PMID: 37626909 PMC10453550

[ref149] Nichols J. A. Nichols A. R. Smirnakis S. M. Engineer N. D. Kilgard M. P. Atzori M. (2011). Vagus nerve stimulation modulates cortical synchrony and excitability through the activation of muscarinic receptors. Neuroscience 189, 207–214. doi: 10.1016/j.neuroscience.2011.05.024, PMID: 21627982

[ref150] Niijima A. (1996). The afferent discharges from sensors for interleukin 1*β* in the hepatoportal system in the anesthetized rat. J. Auton. Nerv. Syst. 61, 287–291. doi: 10.1016/S0165-1838(96)00098-7, PMID: 8988487

[ref151] O’Leary O. F. Ogbonnaya E. S. Felice D. Levone B. R. Conroy C. Fitzgerald P.. (2018). The vagus nerve modulates BDNF expression and neurogenesis in the hippocampus. Eur. Neuropsychopharmacol. 28, 307–316. doi: 10.1016/j.euroneuro.2017.12.004, PMID: 29426666

[ref152] Obukohwo O. M. Ben-Azu B. Nwangwa E. K. Ohwin E. P. Igweh J. C. Adeogun A. E. (2024). Adverse hematological profiles associated with chlorpromazine antipsychotic treatment in male rats: preventive and reversal mechanisms of taurine and coenzyme-Q10. Toxicol. Rep. 12, 448–462. doi: 10.1016/j.toxrep.2024.04.004 38693965 PMC11061245

[ref153] Olsen L. K. Moore R. J. Bechmann N. A. Ethridge V. T. Gargas N. M. Cunningham S. D.. (2022). Vagus nerve stimulation-induced cognitive enhancement: hippocampal neuroplasticity in healthy male rats. Brain Stimul. 15, 1101–1110. doi: 10.1016/j.brs.2022.08.001, PMID: 35970317

[ref154] Onodera J. Nagata H. Nakashima A. Ikegaya Y. Koyama R. (2021). Neuronal brain-derived neurotrophic factor manipulates microglial dynamics. Glia 69, 890–904. doi: 10.1002/glia.23934, PMID: 33119934

[ref155] Ormel P. R. Böttcher C. Gigase F. A. J. Missall R. D. van Zuiden W. Fernández Zapata M. C.. (2020). A characterization of the molecular phenotype and inflammatory response of schizophrenia patient-derived microglia-like cells. Brain Behav. Immun. 90, 196–207. doi: 10.1016/j.bbi.2020.08.012, PMID: 32798663

[ref156] Paolicelli R. C. Sierra A. Stevens B. Tremblay M. E. Aguzzi A. Ajami B.. (2022). Microglia states and nomenclature: a field at its crossroads. Neuron 110, 3458–3483. doi: 10.1016/j.neuron.2022.10.020, PMID: 36327895 PMC9999291

[ref157] Parkhurst C. N. Yang G. Ninan I. Savas J. N. Yates J. R. Lafaille J. J.. (2013). Microglia promote learning-dependent synapse formation through brain-derived neurotrophic factor. Cell 155, 1596–1609. doi: 10.1016/j.cell.2013.11.030, PMID: 24360280 PMC4033691

[ref158] Pavlov V. A. Tracey K. J. (2004). Neural regulators of innate immune responses and inflammation. Cell Mol Life Sci. 61, 2322–2331. doi: 10.1007/s00018-004-4102-3 15378203 PMC11138906

[ref159] Pavlov V. A. Tracey K. J. (2012). The vagus nerve and the inflammatory reflex—linking immunity and metabolism. Nat. Rev. Endocrinol. 8, 743–754. doi: 10.1038/nrendo.2012.189, PMID: 23169440 PMC4082307

[ref160] Pavlov V. A. Wang H. Czura C. J. Friedman S. G. Tracey K. J. (2003). The cholinergic anti-inflammatory pathway: a missing link in Neuroimmunomodulation. Mol. Med. 9, 125–134. doi: 10.1007/BF03402177, PMID: 14571320 PMC1430829

[ref161] Perez S. M. Carreno F. R. Frazer A. Lodge D. J. (2014). Vagal nerve stimulation reverses aberrant dopamine system function in the Methylazoxymethanol acetate rodent model of schizophrenia. J. Neurosci. 34, 9261–9267. doi: 10.1523/JNEUROSCI.0588-14.2014, PMID: 25009259 PMC4087206

[ref162] Picciotto M. R. Higley M. J. Mineur Y. S. (2012). Acetylcholine as a neuromodulator: cholinergic signaling shapes nervous system function and behavior. Neuron 76, 116–129. doi: 10.1016/j.neuron.2012.08.036 23040810 PMC3466476

[ref163] Price C. J. Hoyda T. D. Ferguson A. V. (2008). The area postrema: a brain monitor and integrator of systemic autonomic state. Neuroscientist 14, 182–194. doi: 10.1177/1073858407311100, PMID: 18079557

[ref164] Rodriguez J. I. Kern J. K. (2011). Evidence of microglial activation in autism and its possible role in brain underconnectivity. Neuron Glia Biol. 7, 205–213. doi: 10.1017/S1740925X12000142, PMID: 22874006 PMC3523548

[ref165] Rodríguez-Gómez J. A. Kavanagh E. Engskog-Vlachos P. Engskog M. K. R. Herrera A. J. Espinosa-Oliva A. M.. (2020). Microglia: agents of the CNS pro-inflammatory response. Cells 9:1717. doi: 10.3390/cells9071717 32709045 PMC7407646

[ref166] Roosevelt R. W. Smith D. C. Clough R. W. Jensen R. A. Browning R. A. (2006). Increased extracellular concentrations of norepinephrine in cortex and Hippocampus following Vagus nerve stimulation in the rat. Brain Res. 1119, 124–132. doi: 10.1016/j.brainres.2006.08.048, PMID: 16962076 PMC1751174

[ref167] Rosas-Ballina M. Olofsson P. S. Ochani M. Valdés-Ferrer S. I. Levine Y. A. Reardon C.. (2011). Acetylcholine-synthesizing T cells relay neural signals in a Vagus nerve circuit. Science (New York, N.Y.) 334, 98–101. doi: 10.1126/science.1209985, PMID: 21921156 PMC4548937

[ref168] Ruffoli R. Giorgi F. S. Pizzanelli C. Murri L. Paparelli A. Fornai F. (2011). The chemical neuroanatomy of vagus nerve stimulation. J. Chem. Neuroanat. 42, 288–296. doi: 10.1016/j.jchemneu.2010.12.002, PMID: 21167932

[ref169] Sabers A. Aumüller-Wagner S. Christensen L. R. Henning O. Kostov K. Lossius M.. (2021). Feasibility of transcutaneous auricular vagus nerve stimulation in treatment of drug resistant epilepsy: a multicenter prospective study. Epilepsy Res. 177:106776. doi: 10.1016/j.eplepsyres.2021.106776, PMID: 34597958

[ref170] Savage J. C. St-Pierre M. K. Carrier M. El Hajj H. Novak S. W. Sanchez M. G.. (2020). Microglial physiological properties and interactions with synapses are altered at presymptomatic stages in a mouse model of Huntington’s disease pathology. J. Neuroinflammation 17:98. doi: 10.1186/s12974-020-01782-9 32241286 PMC7118932

[ref171] Sciberras E. Streatfeild J. Ceccato T. Pezzullo L. Scott J. G. Middeldorp C. M.. (2022). Social and economic costs of attention-deficit/hyperactivity disorder across the lifespan. J. Atten. Disord. 26, 72–87. doi: 10.1177/1087054720961828, PMID: 33047627

[ref172] Sgritta M. Dooling S. W. Buffington S. A. Momin E. N. Francis M. B. Britton R. A.. (2019). Mechanisms underlying microbial-mediated changes in social behavior in mouse models of autism Spectrum disorder. Neuron 101, 246–259.e6. doi: 10.1016/j.neuron.2018.11.018, PMID: 30522820 PMC6645363

[ref173] Shah A. P. Carreno F. R. Wu H. Chung Y. A. Frazer A. (2016). Role of TrkB in the anxiolytic-like and antidepressant-like effects of vagal nerve stimulation: comparison with Desipramine. Neuroscience 322, 273–286. doi: 10.1016/j.neuroscience.2016.02.024, PMID: 26899129 PMC4817548

[ref174] Shansky R. M. Lipps J. (2013). Stress-induced cognitive dysfunction: hormone-neurotransmitter interactions in the prefrontal cortex. Front. Hum. Neurosci. 7:123. doi: 10.3389/fnhum.2013.00123 23576971 PMC3617365

[ref175] Shigemoto-Mogami Y. Hoshikawa K. Goldman J. E. Sekino Y. Sato K. (2014). Microglia enhance neurogenesis and oligodendrogenesis in the early postnatal subventricular zone. J. Neurosci. 34, 2231–2243. doi: 10.1523/JNEUROSCI.1619-13.2014, PMID: 24501362 PMC3913870

[ref176] Shivaswamy T. Souza R. R. Engineer C. T. McIntyre C. K. (2022). Vagus nerve stimulation as a treatment for fear and anxiety in individuals with autism Spectrum disorder. J. Psychiatry Brain Sci. 7:e220007. doi: 10.20900/jpbs.20220007 PMC9600938 36303861

[ref177] Silberstein S. D. Calhoun A. H. Lipton R. B. Grosberg B. M. Cady R. K. Dorlas S.. (2016). Chronic migraine headache prevention with noninvasive vagus nerve stimulation. Neurology 87, 529–538. doi: 10.1212/WNL.0000000000002918 27412146 PMC4970666

[ref178] Smucny J. Visani A. Tregellas J. R. (2015). Could Vagus nerve stimulation target hippocampal hyperactivity to improve cognition in schizophrenia? Front. Psych. 6:43. doi: 10.3389/fpsyt.2015.00043 PMC4371554 25852579

[ref179] Snead O. C. (1983). On the sacred disease: the neurochemistry of epilepsy. Int. Rev. Neurobiol. 24, 93–180. doi: 10.1016/S0074-7742(08)60221-4 6140245

[ref180] Snell R. S. (2010). Clinical neuroanatomy. Philadelphia, PA: Wolters Kluwer Health/Lippincott Williams & Wilkins.

[ref181] Starnes K. Miller K. Wong-Kisiel L. Lundstrom B. N. (2019). A review of Neurostimulation for epilepsy in pediatrics. Brain Sci. 9:283. doi: 10.3390/brainsci9100283, PMID: 31635298 PMC6826633

[ref182] Suarez A. N. Hsu T. M. Liu C. M. Noble E. E. Cortella A. M. Nakamoto E. M.. (2018). Gut vagal sensory signaling regulates hippocampus function through multi-order pathways. Nat. Commun. 9:2181. doi: 10.1038/s41467-018-04639-1 29872139 PMC5988686

[ref183] Suller Marti A. Mirsattari S. M. MacDougall K. Steven D. A. Parrent A. de Ribaupierre S.. (2020). Vagus nerve stimulation in patients with therapy-resistant generalized epilepsy. Epilepsy Behav. 111:107253. doi: 10.1016/j.yebeh.2020.107253, PMID: 32615417

[ref184] Tang H. Li J. Zhou Q. Li S. Xie C. Niu L.. (2022). Vagus nerve stimulation alleviated cerebral ischemia and reperfusion injury in rats by inhibiting pyroptosis via α7 nicotinic acetylcholine receptor. Cell Death Discov. 8:54. doi: 10.1038/s41420-022-00852-6 35136042 PMC8825823

[ref185] Taylor N. L. D’Souza A. Munn B. R. Lv J. Zaborszky L. Müller E. J.. (2022). Structural connections between the noradrenergic and cholinergic system shape the dynamics of functional brain networks. NeuroImage 260:119455. doi: 10.1016/j.neuroimage.2022.119455, PMID: 35809888 PMC10114918

[ref186] Tian X. Liu H. Xiang F. Xu L. Dong Z. (2019). β-Caryophyllene protects against ischemic stroke by promoting polarization of microglia toward M2 phenotype via the TLR4 pathway. Life Sci. 237:116915. doi: 10.1016/j.lfs.2019.116915, PMID: 31610207

[ref187] Tregellas J. R. Wylie K. P. (2018). Alpha7 nicotinic receptors as therapeutic targets in schizophrenia. Nicotine Tob. Res. 21, 349–356. doi: 10.1093/ntr/nty034 PMC6379034 30137618

[ref188] Tremblay M. È. (2020). A diversity of cell types, subtypes and phenotypes in the central nervous system: The importance of studying their complex relationships. Front. Cell. Neurosci. 14:628347. doi: 10.3389/fncel.2020.628347, PMID: 33424557 PMC7785523

[ref189] Tremblay M. È. (2021). Microglial functional alteration and increased diversity in the challenged brain: insights into novel targets for intervention. Brain Behav. Immun. Health 16:100301. doi: 10.1016/j.bbih.2021.100301, PMID: 34589793 PMC8474548

[ref190] Ueno M. Fujita Y. Tanaka T. Nakamura Y. Kikuta J. Ishii M.. (2013). Layer V cortical neurons require microglial support for survival during postnatal development. Nat. Neurosci. 16, 543–551. doi: 10.1038/nn.3358, PMID: 23525041

[ref191] van Os J. Kapur S. (2009). Schizophrenia. Lancet 374, 635–645. doi: 10.1016/S0140-6736(09)60995-8, PMID: 19700006

[ref192] Véronneau-Veilleux F. Robaey P. Ursino M. Nekka F. (2022). A mechanistic model of ADHD as resulting from dopamine phasic/tonic imbalance during reinforcement learning. Front. Comput. Neurosci. 16:16. Available from: doi: 10.3389/fncom.2022.849323 PMC9342605 35923915

[ref193] Wang L. Chang X. She L. Xu D. Huang W. Poo M.. (2015). Autocrine action of BDNF on dendrite development of adult-born hippocampal neurons. J. Neurosci. 35, 8384–8393. doi: 10.1523/JNEUROSCI.4682-14.2015, PMID: 26041908 PMC6605324

[ref194] Wang M. X. Wumiti A. Zhang Y. W. Gao X. S. Huang Z. Zhang M. F.. (2023). Transcutaneous cervical vagus nerve stimulation improved motor cortex excitability in healthy adults: a randomized, single-blind, self-crossover design study. Front. Neurosci. 17:17. doi: 10.3389/fnins.2023.1234033 PMC10579560 37854293

[ref195] Wang G. J. Yang J. Volkow N. D. Telang F. Ma Y. Zhu W.. (2006). Gastric stimulation in obese subjects activates the hippocampus and other regions involved in brain reward circuitry. Proc. Natl. Acad. Sci. USA 103, 15641–15645. doi: 10.1073/pnas.0601977103 17023542 PMC1592230

[ref196] Wang Z. Yuan X. Zhang Q. Wen J. Cheng T. Qin X.. (2022). Effects of stable Vagus nerve stimulation efficacy on autistic behaviors in ten pediatric patients with drug resistant epilepsy: An observational study. Front. Pediatr. 10:846301. doi: 10.3389/fped.2022.846301 35311037 PMC8924444

[ref197] Wang Y. Zhan G. Cai Z. Jiao B. Zhao Y. Li S.. (2021). Vagus nerve stimulation in brain diseases: therapeutic applications and biological mechanisms. Neurosci. Biobehav. Rev. 127, 37–53. doi: 10.1016/j.neubiorev.2021.04.018, PMID: 33894241

[ref198] Watters J. J. Pocock J. M. (2014). “Microglial physiology” in Microglia in health and disease. eds. Tremblay M. È. Sierra A. (New York, NY: Springer), 47–79.

[ref199] Wiles C. C. R. Wrigley B. Greene J. R. T. (2007). Re-examination of the medullary rootlets of the accessory and vagus nerves. Clin. Anat. 20, 19–22. doi: 10.1002/ca.20260, PMID: 16317753

[ref200] Xia X.-M. Duan Y. Wang Y.-P. Han R.-X. Dong Y.-F. Jiang S.-Y.. (2024). Vagus nerve stimulation as a promising neuroprotection for ischemic stroke via α7nAchR-dependent inactivation of microglial NLRP3 inflammasome. Acta Pharmacol. Sin. 45, 1349–1365. doi: 10.1038/s41401-024-01245-4 38504011 PMC11192746

[ref201] Xie H. Ma J. Ji T. Liu Q. Cai L. Wu Y. (2022). Vagus nerve stimulation in children with drug-resistant epilepsy of monogenic etiology. Front. Neurol. 13:13. doi: 10.3389/fneur.2022.951850 PMC9475310 36119689

[ref202] Yakunina N. Kim S. S. Nam E. C. (2017). Optimization of transcutaneous Vagus nerve stimulation using functional MRI. Neuromodulation: Technology at the Neural. Interface 20, 290–300. doi: 10.1111/ner.12541 27898202

[ref203] Yang T. Nie Z. Shu H. Kuang Y. Chen X. Cheng J.. (2020). The Role of BDNF on neural plasticity in depression. Front. Cell. Neurosci. 14:82. doi: 10.3389/fncel.2020.00082 32351365 PMC7174655

[ref204] Yao X. Liu S. Ding W. Yue P. Jiang Q. Zhao M.. (2017). TLR4 signal ablation attenuated neurological deficits by regulating microglial M1/M2 phenotype after traumatic brain injury in mice. J. Neuroimmunol. 310, 38–45. doi: 10.1016/j.jneuroim.2017.06.006, PMID: 28778443

[ref205] Yap J. Y. Y. Keatch C. Lambert E. Woods W. Stoddart P. R. Kameneva T. (2020). Critical review of transcutaneous Vagus nerve stimulation: challenges for translation to clinical practice. Front. Neurosci. 14:14. doi: 10.3389/fnins.2020.00284 32410932 PMC7199464

[ref206] Yuan H. Silberstein S. D. (2016). Vagus nerve and Vagus nerve stimulation, a comprehensive review: part III. Headache: The journal of head and face. Pain 56, 479–490. doi: 10.1111/head.12649 26364805

[ref207] Zabara J. (1992). Inhibition of experimental seizures in canines by repetitive vagal stimulation. Epilepsia 33, 1005–1012. doi: 10.1111/j.1528-1157.1992.tb01751.x, PMID: 1464256

[ref208] Zaehle T. Krauel K. (2021). Transcutaneous vagus nerve stimulation in patients with attention-deficit/hyperactivity disorder: a viable option? Prog. Brain Res. 264, 171–190. doi: 10.1016/bs.pbr.2021.03.001, PMID: 34167655

[ref209] Zhang J. Geula C. Lu C. Koziel H. Hatcher L. M. Roisen F. J. (2003). Neurotrophins regulate proliferation and survival of two microglial cell lines *in vitro*. Exp. Neurol. 183, 469–481. doi: 10.1016/S0014-4886(03)00222-X, PMID: 14552887

[ref210] Zhang P. Han D. Tang T. Zhang X. Dai K. (2008). Inhibition of the development of collagen-induced arthritis in Wistar rats through vagus nerve suspension: a 3-month observation. Inflamm. Res. 57, 322–328. doi: 10.1007/s00011-008-8070-1 18607536

[ref211] Zhang L. Liu Y. Wang S. Long L. Zang Q. Ma J.. (2021). Vagus nerve stimulation mediates microglia M1/2 polarization via inhibition of TLR4 pathway after ischemic stroke. Biochem. Biophys. Res. Commun. 577, 71–79. doi: 10.1016/j.bbrc.2021.09.004, PMID: 34507068

[ref212] Zhang W. Mou Z. Zhong Q. Liu X. Yan L. Gou L.. (2024). Transcutaneous auricular vagus nerve stimulation improves social deficits through the inhibition of IL-17a signaling in a mouse model of autism. Front. Psychiatry 15:15. doi: 10.3389/fpsyt.2024.1393549 PMC11237520 38993386

[ref213] Zhang H. Zhao H. Zeng C. Van Dort C. Faingold C. L. Taylor N. E.. (2018). Optogenetic activation of 5-HT neurons in the dorsal raphe suppresses seizure-induced respiratory arrest and produces anticonvulsant effect in the DBA/1 mouse SUDEP model. Neurobiol. Dis. 110, 47–58. doi: 10.1016/j.nbd.2017.11.003, PMID: 29141182 PMC5748009

[ref214] Zhao X. P. Zhao Y. Qin X. Y. Wan L. Y. Fan X. X. (2019). Non-invasive Vagus nerve stimulation protects against cerebral ischemia/reperfusion injury and promotes microglial M2 polarization via interleukin-17A inhibition. J. Mol. Neurosci. 67, 217–226. doi: 10.1007/s12031-018-1227-7, PMID: 30484061

[ref215] Zhou L. Lin J. Lin J. Kui G. Zhang J. Yu Y. (2014). Neuroprotective effects of vagus nerve stimulation on traumatic brain injury. Neural Regen. Res. 9, 1585–1591. doi: 10.4103/1673-5374.141783, PMID: 25368644 PMC4211199

[ref216] Zhu S. Huang S. Xia G. Wu J. Shen Y. Wang Y.. (2021). Anti-inflammatory effects of α7-nicotinic ACh receptors are exerted through interactions with adenylyl cyclase-6. Br. J. Pharmacol. 178, 2324–2338. doi: 10.1111/bph.15412, PMID: 33598912

[ref217] Zhuo C. Tian H. Song X. Jiang D. Chen G. Cai Z.. (2023). Microglia and cognitive impairment in schizophrenia: translating scientific progress into novel therapeutic interventions. Schizophr 9, 1–8. doi: 10.1038/s41537-023-00370-z PMC10333203 37429882

